# Supplementary material for: Contrasting Effects of Local Environmental and Biogeographic Factors on the Composition and Structure of Bacterial Communities in Arid Monospecific Mangrove Soils
Source: Microbiol Spectr. 2022 Jan 5;10(1):e00903-21. doi: 10.1128/spectrum.00903-21 (PMC8729789; doi:10.1128/spectrum.00903-21)
Supplement: SUPPLEMENTAL FILE 1 — Supplemental material. Download SPECTRUM00903-21_Supp_1_seq7.pdf, PDF file, 4.9 MB [file spectrum00903-21_supp_1_seq7.pdf]

Supplementary Information Workflow

Contrasting effects of local environmental and biogeographic factors on the composition and structure of bacterial communities in arid monospecific mangrove soils

Timothy Thomson, Fusi, M., Bennett-Smith, M.F., Prinz, N., Aylagas, E., Carvalho, S., Lovelock, C.E., Jones, B.H., Ellis, J.I.

11/11/2021

This workflow document is designed to provide the highest amount of transparency and reproducibility for the reader. The analyses herein can be run with the same data set or with another set of data, if required. There are two sets of figures in this document. The figures produced for and published in the connected manuscript "Contrasting effects of local environmental and biogeographic factors on the composition and structure of bacterial communities in arid monospecific mangrove soils" which ale captioned with the figure numbers they hold in the manuscript, and with Supplementary figures, which are consecutively labelled with 'Supplementary Figure No' throughout this markdown document.

Loading required packages

```
library(devtools)
library(dada2)
library(vegan)
library(plyr)
library(dplyr)
library(tibble)
library(data.table)
library(pairwiseAdonis)
library(phyloseq)
library(ggplot2)
library(gridExtra)
library(corrplot)
library(RColorBrewer)
library(viridis)
library(tidyverse)
library(reshape2)
library(metagMisc)
library(Hmisc)
library(MASS)
library(DESeq2)
library(Tax4Fun2)
library(seqinr)
library(ggpubr)
library(phangorn)
library(ape)
library(picante)
library(DECIPHER)
library(BBmisc)
```

1. Data import and downstream processing

The raw data (demultiplexed) is accessible for download from the NCBI SRA repository under the accession number PRJNA720541.The dada2 pipeline has been applied as described in the methods section of the

manuscript.

Set your working directory

```
setwd("~/Desktop/SpatialPatterns/sp_data")
```

Read in all neccessary files: abundance table, taxonomy, meta data

```
abund_tab <- readRDS("sp_data_tables/sp_asv_table.rds")
tax_tab <- readRDS("sp_data_tables/sp_taxonomy_table_SILVA.rds")
meta_tab <- read.csv("sp_data_tables/sp_meta_table.csv", row.names = 1, check.names = F)
meta_tab$Depth <- relevel(meta_tab$Depth, "Surface")
```

Furthermore we align all samples to be sure they are all in the same order in asv- and meta-tables.

```
common_ids <- intersect(rownames(meta_tab), rownames(abund_tab))
asv_tab <- abund_tab[common_ids,]
meta_tab <- meta_tab[common_ids,]
```

1.1 Create phyloseq object

We create a phyloseq object consisting of an asv-, a taxonomy-, and a meta data table. The phylosec object is called SC which can be used for further analysis with phyloseq

```
ASV = otu_table(asv_tab, taxa_are_rows = FALSE)
TAX = tax_table(tax_tab)
samples = sample_data(meta_tab)

SP <- phyloseq(ASV, TAX, samples)
SP
```

```
## phyloseq-class experiment-level object
## otu_table()   OTU Table:         [ 91152 taxa and 120 samples ]
## sample_data() Sample Data:       [ 120 samples by 5 sample variables ]
## tax_table()   Taxonomy Table:    [ 91152 taxa by 6 taxonomic ranks ]
```

1.2 Filter out artefacts

First we can filter out artifacts that are left over from extraction and sequencing by selecting only ASVs that belong to the Bacteria kingdom, and none that are grouped in the 'Order' of Chloroplasts or the 'Family' of Mitochondria. We then extract the tables to save them independently in case we want to return to this level of filtration.

```
sp_bact <- subset_taxa(SP, Kingdom == "Bacteria" &
                              Order %nin% "Chloroplast" & Family %nin% "Mitochondria")


asv_sp_bact <- data.frame(otu_table(sp_bact))
tax_sp_bact <- data.frame(tax_table(sp_bact))

write.csv(asv_sp_bact, "sp_data_tables/asv_aligned_lowseqremoved.csv")
write.csv(tax_sp_bact, "sp_data_tables/tax_aligned_lowseqremoved.csv")
```

Now we remove ASVs that are below a prevalence threshold that we set to 5% of all samples. That means, that ASVs appearing in less than 5% of all samples are being removed. We then extract the tables to save them independently in case we want to return to this level of filtration.

```
sp_filter <- phyloseq_filter_prevalence(sp_bact, prev.trh = 0.05, abund.trh = NULL,
                                        threshold_condition = "OR",
```

```
                                        abund.type = "total")

asv_sp_filter <- data.frame(otu_table(sp_filter))
tax_sp_filter <- data.frame(tax_table(sp_filter))

write.csv(asv_sp_filter, "sp_data_tables/asv_aligned_lowseqremoved_lowprevremoved.csv")
write.csv(tax_sp_filter, "sp_data_tables/tax_aligned_lowseqremoved_lowprevremoved.csv")
```

We then remove two possibly contaminated samples

```
sp_filter_contam_rem = subset_samples(sp_filter,
                                      sample_names(sp_filter) != "KAMW3-3Sub" &
                                        sample_names(sp_filter) != "MBD51SURF")
```

1.3 Test for sequencing depth

1.3.1 Number of reads and reads per sample

We can plot a rank abundance curve and the sequencing depth per sample as a diagnostic tool, as well as the rarefaction curves in the plot below.

```
sp_readsums <- data.frame(nreads = sort(taxa_sums(sp_filter_contam_rem), TRUE),
                          sorted = 1:ntaxa(sp_filter_contam_rem), type = "ASVs")
sp_readsums <- rbind(sp_readsums,
                     data.frame(nreads = sort(sample_sums(sp_filter_contam_rem), TRUE),
                                sorted = 1:nsamples(sp_filter_contam_rem),
                                type = "Samples"))
```

```
p <- ggplot(sp_readsums, aes(x = sorted, y = nreads)) +
  geom_point(stat = "identity")
p + labs(title = "Total number of reads") +
  scale_y_log10() +
  facet_wrap(~type, 1, scales = "free")
```

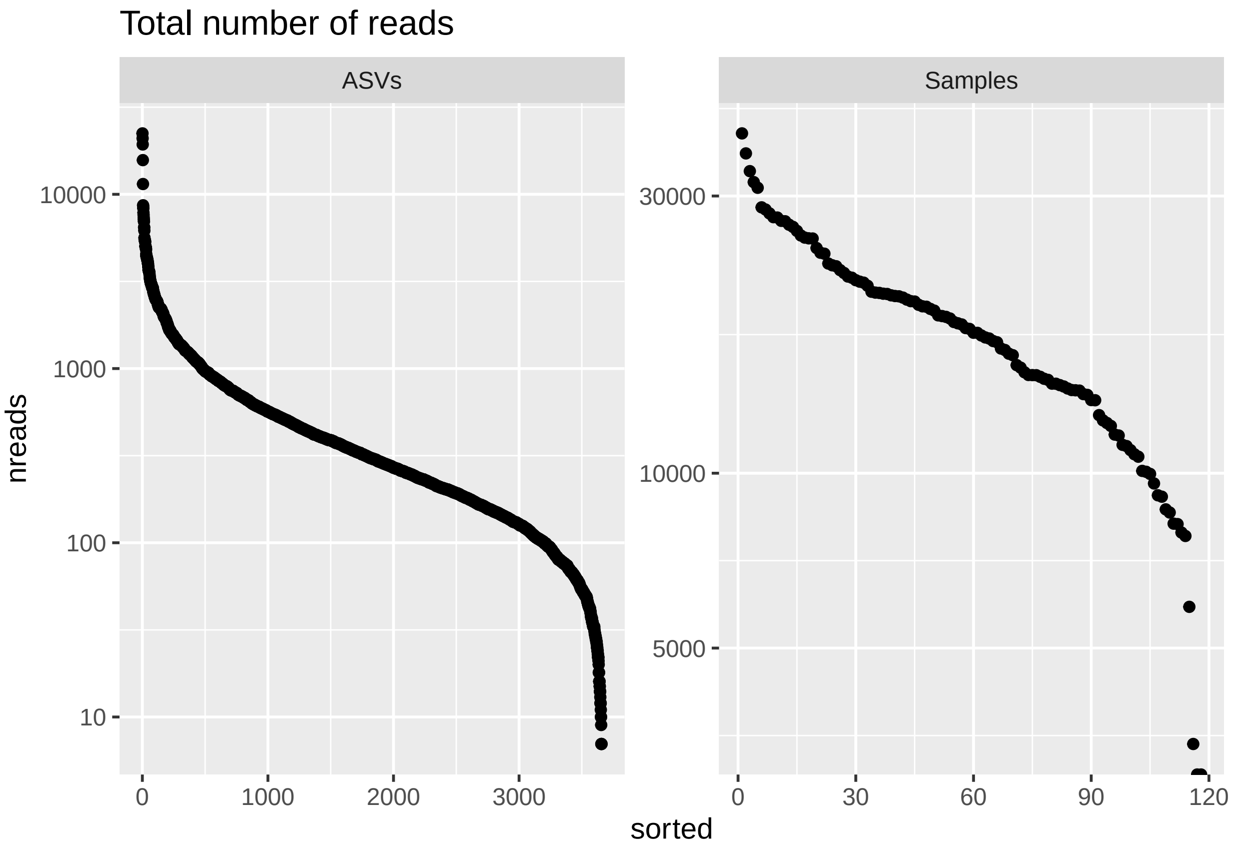

Supplementary Figure S1

Rank-Abundance curves of reads per ASVs (left) and reads per sample (right) as a diagnostic tool for sequencing depth.

1.3.2 Rarefaction curves

```
rarecurve(asv_sp_filter, step = 1000,
          col = brewer.pal(n = 8, name = "Dark2"),
          lty = c(1:4), label = FALSE,
          main = "Rarefaction curves")
```

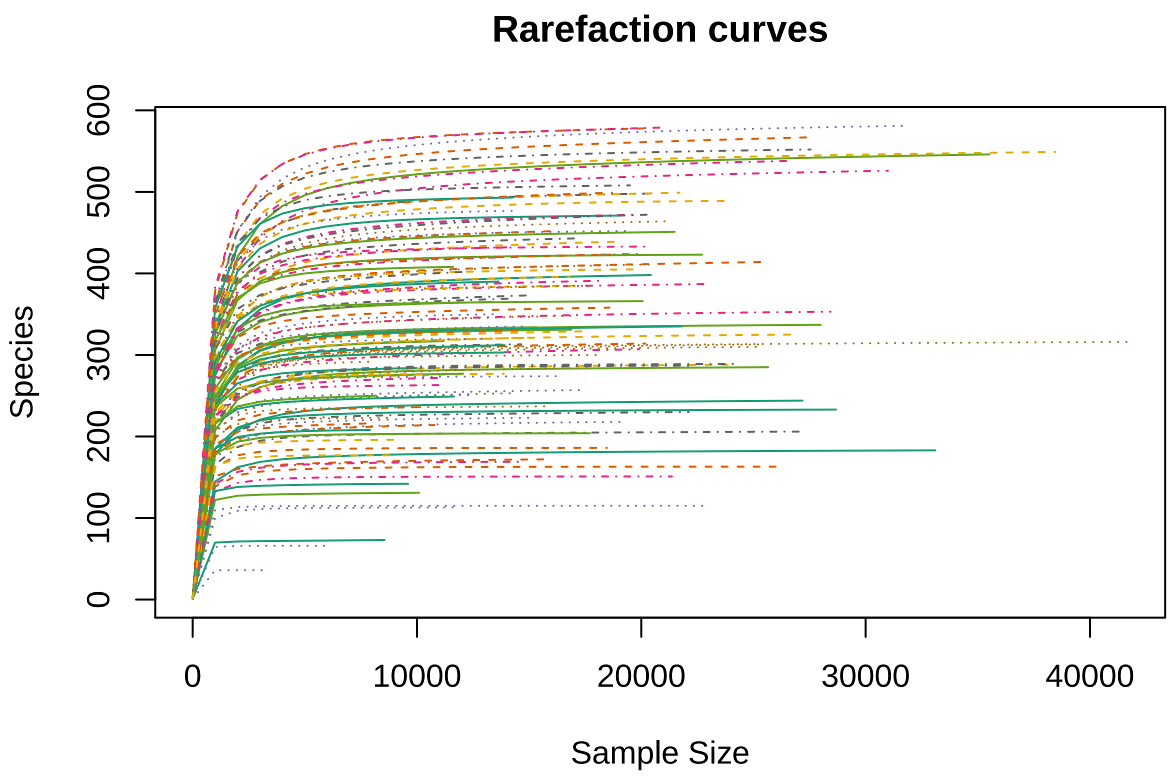

Supplementary Figure S2

Rarefaction curves of each sample indicating the number of ASVs and the threshold at which a sufficient sequencing depth was reached to capture the 'true' diversity present in the sample. Each line represents one sample and the colours and line types were arbitraily chosen for better differentiation between them.

Pruning

We then remove samples with too low sequence coverage (here chosen to be any sample with less than 5000 sequences). Since we later rarify the data to an even sequencing depth, this step isn't totally necessary. However, I do it here to also have a "clean" non-rarified version of the data.

```
sp_pruned <- prune_samples(sample_sums(sp_filter_contam_rem) > 5000,
                           sp_filter_contam_rem)
```

The last step of data filtering is to rarify the samples to an even sequencing depth. Here we choose the minimum sequencing depth as the cutoff, which we can do since we have removed all samples below 5000 sequences. We then extract the tables to save them independently in case we want to work with this level of filtration.

```
set.seed(33)
sp_rar <- rarefy_even_depth(sp_pruned, sample.size = min(sample_sums(sp_pruned)),
                            rngseed = TRUE, trimOTUs = TRUE, verbose = TRUE)
```

Supplementary Table S1

Phyloseq object describing the number of ASVs across all 115 samples.

```
sp_rar
```

```
## phyloseq-class experiment-level object
## otu_table()   OTU Table:         [ 3657 taxa and 115 samples ]
```

```
## sample_data() Sample Data:       [ 115 samples by 5 sample variables ]
## tax_table()   Taxonomy Table:    [ 3657 taxa by 6 taxonomic ranks ]
```

```
asv_sp_rar <- data.frame(otu_table(sp_rar))
tax_sp_rar <- data.frame(tax_table(sp_rar))
```

1.4 Save relevant tables

Save trimmed ASV table with sequences for phylogenetic processing.

```
asv_sp_rar_seqs <- asv_sp_rar

write.csv(asv_sp_rar_seqs, "sp_data_tables/asv_sp_rarified_with_sequences.csv")
```

This step is needed after the DADA2 pipeline, since all sequences are still in the column names. It changes colnames of otu_tab and row names of tax_tab to ASV#

```
seqids <- c(1:length(colnames(asv_sp_rar)))
seqids <- paste0("ASV",seqids)
asv_tab <- abund_tab
colnames(asv_sp_rar) <- seqids
row.names(tax_sp_rar) <- seqids
```

Save Sequence table and taxonomy table

```
write.csv(asv_sp_rar, "sp_data_tables/asv_sp_rarefied.csv")
write.csv(tax_sp_rar, "sp_data_tables/tax_sp_rarefied.csv")
```

Since some samples were discarded, we save the trimmed meta file as well.

```
meta_sp_trimmed <- data.frame(sample_data(sp_rar))
meta_sp_trimmed$Depth <- ordered(meta_sp_trimmed$Depth,
                                 levels = c("Surface", "Subsurface"))
write.csv(meta_sp_trimmed, "sp_data_tables/meta_sp_trimmed.csv")
```

To work with the filtered and trimmed phyloseq object we use "sp_rar". The individual data tables from this object are called "asv_sp_rar", "tax_sp_rar", and "meta_sp_trimmed". These can be used for analyses outside of phyloseq.

1.5 Aglomeration to different taxonomic levels

Supplementary Table S2

Phyloseq object describing the number of phyla [[1]] and genera [[2]] across all 115 samples.

```
phyl <- tax_glom(sp_rar, "Phylum")
gen <- tax_glom(sp_rar, "Genus")
print(c(phyl, gen))
```

```
## [[1]]
## phyloseq-class experiment-level object
## otu_table()   OTU Table:         [ 37 taxa and 115 samples ]
## sample_data() Sample Data:       [ 115 samples by 5 sample variables ]
## tax_table()   Taxonomy Table:    [ 37 taxa by 6 taxonomic ranks ]
##
## [[2]]
## phyloseq-class experiment-level object
## otu_table()   OTU Table:         [ 233 taxa and 115 samples ]
## sample_data() Sample Data:       [ 115 samples by 5 sample variables ]
```

```
## tax_table()   Taxonomy Table:    [ 233 taxa by 6 taxonomic ranks ]
```

Extract and save to file

```
phyl_sp <- data.frame(otu_table(phyl))
gen_sp <- data.frame(otu_table(gen))

write.csv(phyl_sp, "sp_data_tables/phyl_sp.csv")
write.csv(gen_sp, "sp_data_tables/gen_sp.csv")
```

2. Rank-Abundance

We can plot the Rank-Abundance relationship of the filtered and pruned data to investigate the impact that abundant and rare species may have on the dataset.

```
asv_sp_rar_t <- as.data.frame(t(asv_sp_rar))
asv_sp_rar_t$ASV <- row.names(asv_sp_rar_t)
long_asv <- melt(asv_sp_rar_t, id = "ASV")
names(long_asv)[2] <- "ID"
names(long_asv)[3] <- "Abundance"
meta_sp_trimmed$Label <- row.names(meta_sp_trimmed)
long_all <- merge(long_asv, meta_sp_trimmed, by.x = "ID", by.y = "Label")
```

```
ggplot(ra_ind, aes(Rank, Abundance, colour = Depth, shape = Zone))+
  geom_point(alpha = .9, cex = 1.5)+
  geom_line(stat = "identity")+
  scale_colour_manual(values = c("grey74", "grey50"))+
  facet_grid(Exposure~GeographicRegion)+
  scale_x_log10()+
  theme_classic()+
  guides(colour = guide_legend(override.aes = list(size = 2)),
         shape = guide_legend(override.aes = list(size = 2)))+
  theme(panel.border = element_rect(colour = "black", fill = NA))
```

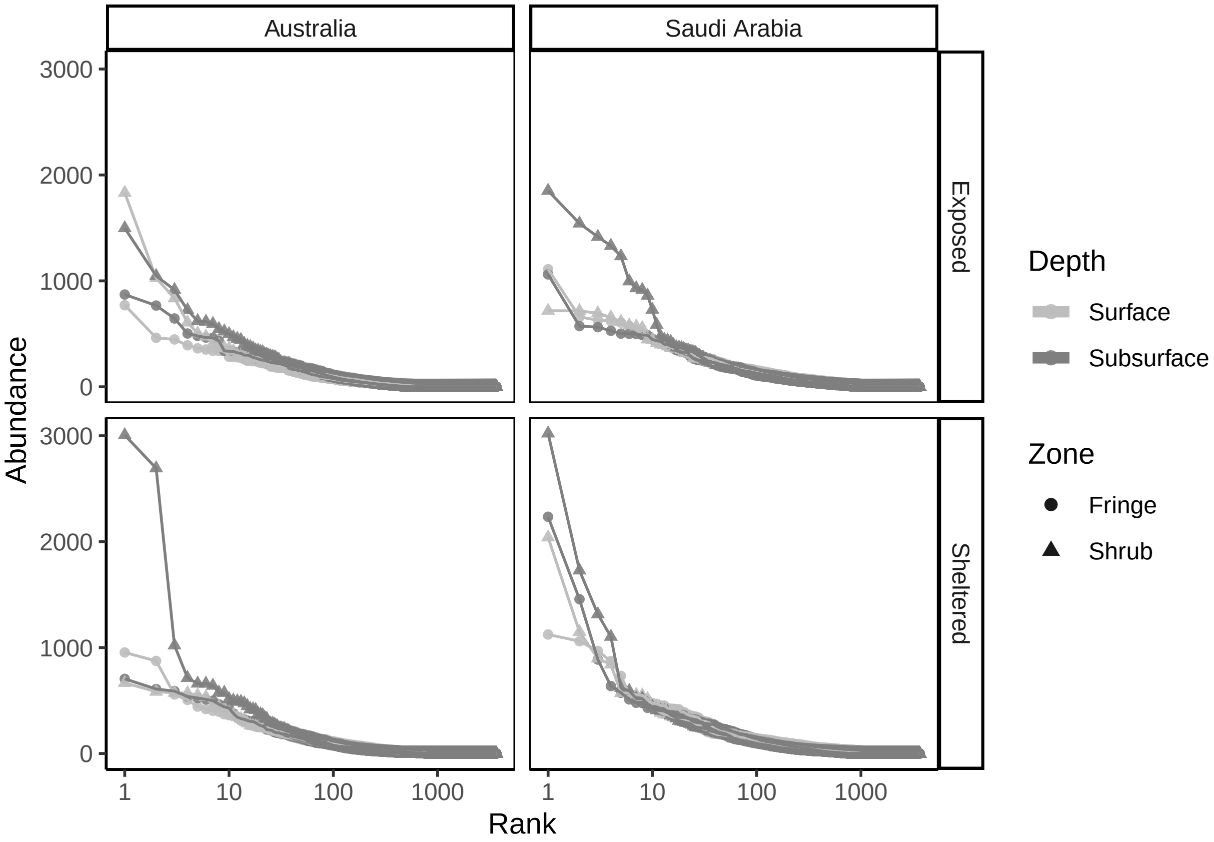

Figure 1A

Rank-Abundance relationship of ASVs between experimental factors.

3. Alpha diversity estimates

We can use phyloseq to calculate richness and diversity estimates, which we add to the meta table. The common measures of "observed richness" and the "shannon diversity index" are calculated.

```
aDiv_est <- estimate_richness(sp_rar, measures = c("Observed","Shannon"))

aDiv <- data.frame(cbind(meta_sp_trimmed, aDiv_est))

write.csv(aDiv, "sp_data_tables/alpha_diversity.csv")
```

To see the table of alpha-diversity metrics created we can use the folloing command.

```
print(aDiv[,c(1, 3:5,7:8)])
```

3.1 Observed species

A visual and a statistical method are applied to test for the normality of the distribution of these estimates. A linear model was created and an analysis of variance (ANOVA) was performed. Furthermore, a post-hoc comparison was applied to disentangle the finer dinamics of differences (not reported on further). There is also a chunk of code to create boxplots of each estimator across all factors.

```
##
##  Shapiro-Wilk normality test
```

```
##
## data:  aDiv$Observed
## W = 0.98838, p-value = 0.4329
```

Supplementary Table S3

Analysis of variance table of observed ASVs indicating significant differences between the interaction factors geographic region, exposure, and zone.

```
aOb4
```

```
## Analysis of Variance Table
##
## Response: Observed
##                                  Df Sum Sq Mean Sq F value    Pr(>F)
## GeographicRegion                  1  63440   63440 10.3741  0.001711 **
## Exposure                          1      0       0  0.0001  0.993031
## Zone                              1  10351   10351  1.6927  0.196151
## Depth                             1  55342   55342  9.0499  0.003302 **
## GeographicRegion:Exposure         1 115261  115261 18.8483 3.318e-05 ***
## GeographicRegion:Zone             1 244429  244429 39.9706 6.715e-09 ***
## Exposure:Zone                     1 110044  110044 17.9950 4.850e-05 ***
## GeographicRegion:Depth            1  31031   31031  5.0744  0.026403 *
## Exposure:Depth                    1   8162    8162  1.3347  0.250643
## Zone:Depth                        1  20750   20750  3.3931  0.068345 .
## GeographicRegion:Exposure:Zone    1  53980   53980  8.8272  0.003694 **
## Residuals                       103 629868    6115
## ---
## Signif. codes:  0 '***' 0.001 '**' 0.01 '*' 0.05 '.' 0.1 ' ' 1
```

Results of the pairwise comparison between observed species across all interaction factors.

```
TukeyHSD(Ob4)
model.tables(Ob4, "mean")
```

```
ggplot(aDiv, aes(Zone, Observed, colour = GeographicRegion, fill = Depth))+
  geom_boxplot(notch = T)+
  scale_fill_manual(values = c("gray90", "grey50"))+
  scale_colour_brewer(palette = "Dark2")+
  labs(y = "Observed ASVs")+
  facet_grid(Exposure~GeographicRegion)+
  theme_bw()
```

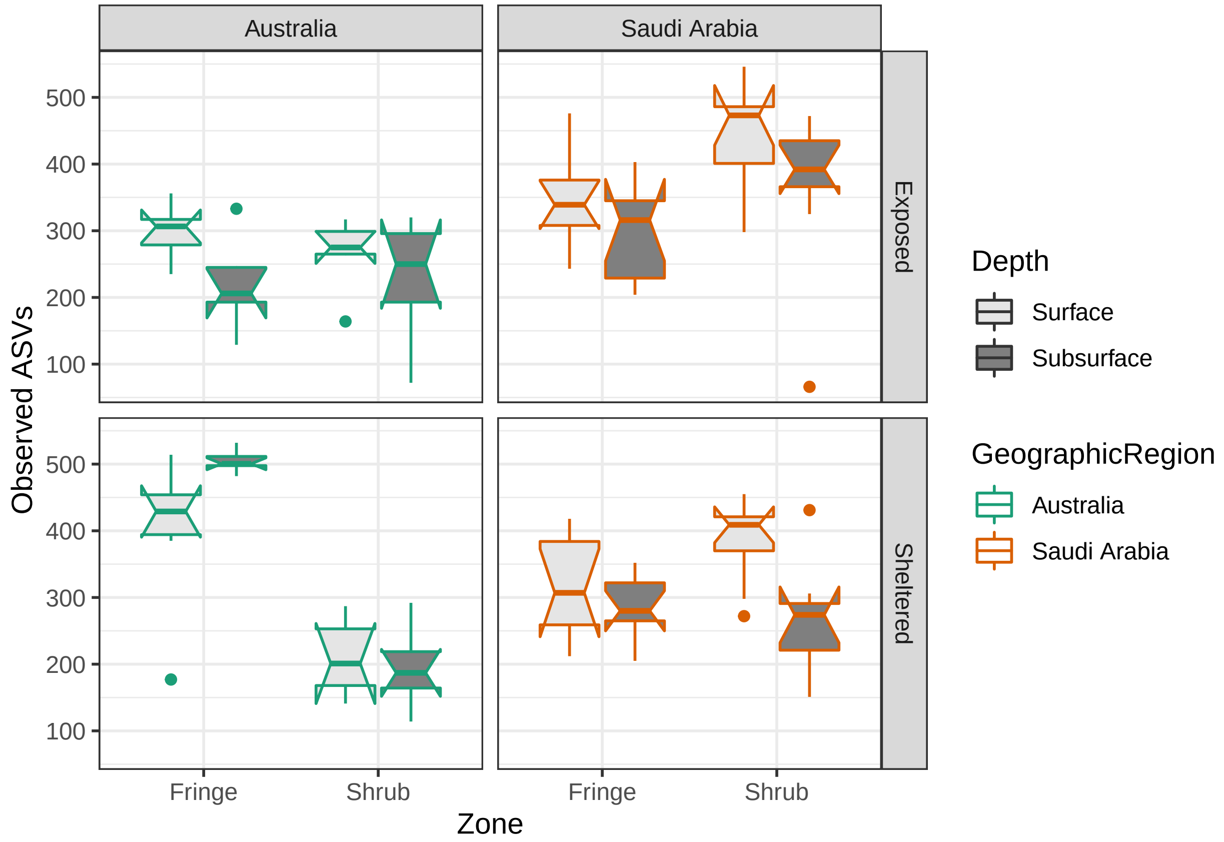

Figure 1B

Species richness described by the number of observed ASVs across all factors. The boxplots indicate the median with the interquartile range (IQR) between the 25th and 75th percentile, and the whiskers extend 1.5xIQR. Boxes were plotted with the notch (+/- 1.58xIQR/sqrt(n)) to display likely statistical significance if notches don't overlap.

3.2 Shannon Diversity

The distribution of the shannon diversity index is not normal but rather skewed to the right. A test of the distribution of the residuals yielded enougth support to advance with an ANOVA nonetheless.

```
##
##  Shapiro-Wilk normality test
##
## data:  aDiv$Shannon
## W = 0.93676, p-value = 3.807e-05
```

Supementary Table S4

Analysis of variance table of Shannon diversity index indicating significant differences between the interaction factors zone and depth.

```
aSha5
```

```
## Analysis of Variance Table
##
## Response: Shannon
##                             Df  Sum Sq Mean Sq F value    Pr(>F)
```

```
## GeographicRegion            1  1.7907 1.79069 17.3535 6.425e-05 ***
## Exposure                    1  0.0204 0.02045  0.1981 0.6571465
## Zone                        1  1.6404 1.64037 15.8967 0.0001245 ***
## Depth                       1  2.1839 2.18394 21.1644 1.193e-05 ***
## GeographicRegion:Exposure   1  1.1393 1.13933 11.0411 0.0012308 **
## GeographicRegion:Zone       1  2.5751 2.57511 24.9552 2.378e-06 ***
## Exposure:Zone               1  0.7209 0.72093  6.9865 0.0094829 **
## GeographicRegion:Depth      1  0.1210 0.12099  1.1725 0.2813957
## Exposure:Depth              1  0.0129 0.01286  0.1246 0.7248119
## Zone:Depth                  1  0.5448 0.54475  5.2791 0.0235845 *
## Residuals                 104 10.7317 0.10319
## ---
## Signif. codes:  0 '***' 0.001 '**' 0.01 '*' 0.05 '.' 0.1 ' ' 1
```

```r
par(mfrow = c(2,2))
plot(Sha5)
```

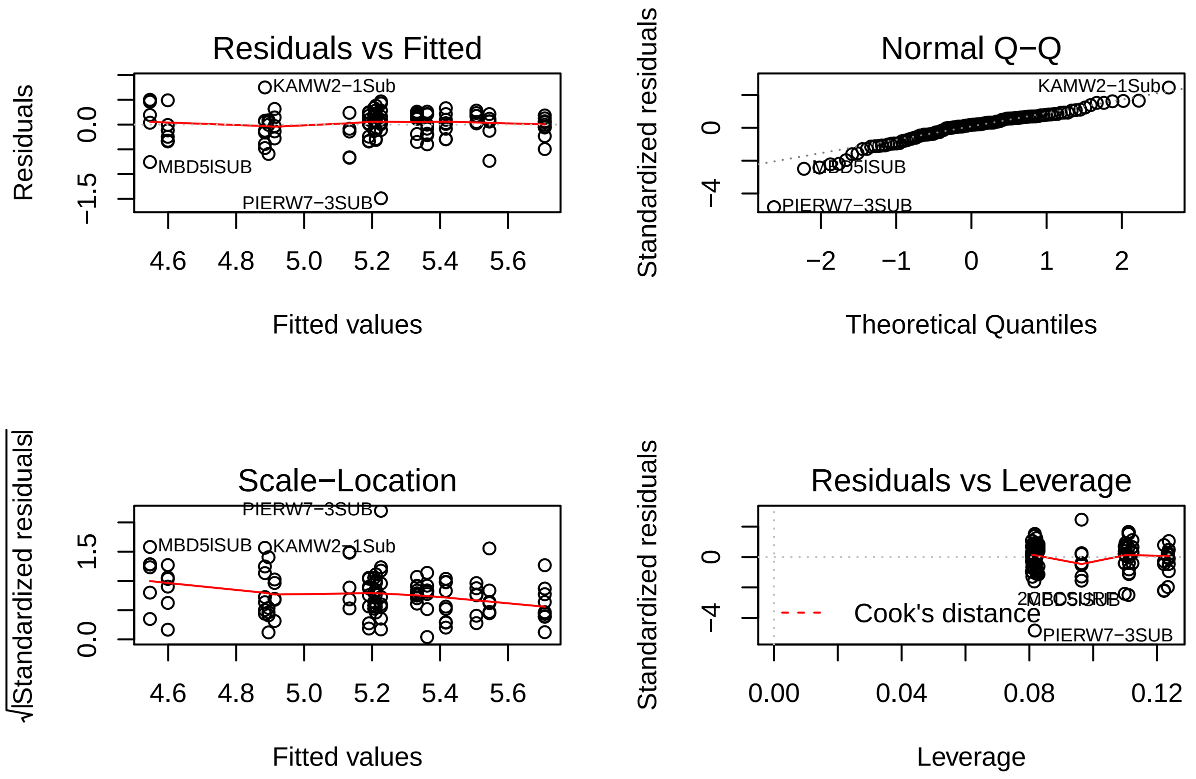


```r
par(mfrow = c(1,1))
```

Diagnostic plots

Diagnostic plots to visually evaluate the fit of the model.

Results of the pairwise comparison between Shannon diversity index across all interaction factors.

```r
TukeyHSD(Sha5)
model.tables(Sha5, "mean")
```

```r
ggplot(aDiv, aes(Zone, Shannon, colour = GeographicRegion,fill = Depth))+
  geom_boxplot(notch = T)+
  scale_fill_manual(values = c("gray90", "grey50"))+
```

```
  scale_colour_brewer(palette = "Dark2")+
  labs(y = "Shannon (H') diversity index")+
  facet_grid(cols = vars(GeographicRegion), rows = vars(Exposure))+
  theme_bw()
```

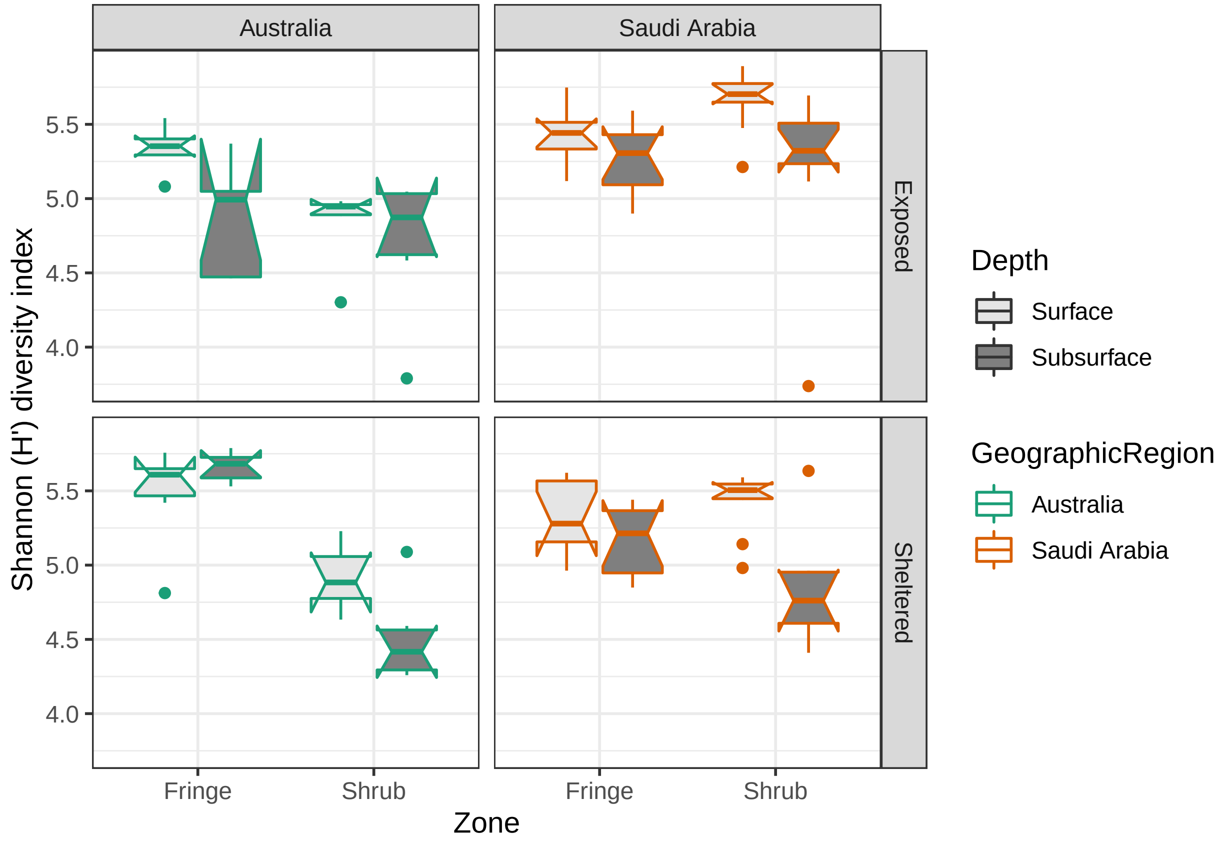

Figure 1C

Shannon diversity index across all experimental factors. The boxplots indicate the median with the interquartile range (IQR) between the 25th and 75th percentile, and the whiskers extend 1.5xIQR. Boxes were plotted with the notch (+/- 1.58xIQR/sqrt(n)) to display likely statistical significance if notches don't overlap.

4. Phylogenetic Diversity

```
seqs <- getSequences(as.matrix(asv_sp_rar_seqs))
names(seqs) <- seqs
alignment <- AlignSeqs(DNAStringSet(seqs), anchor=NA)
```

The phangorn R package is then used to construct a phylogenetic tree. Here we first construct a neighbor-joining tree, and then fit a GTR+G+I (Generalized time-reversible with Gamma rate variation) maximum likelihood tree using the neighbor-joining tree as a starting point.

```
phang.align <- phyDat(as(alignment, "matrix"), type="DNA")
dm <- dist.ml(phang.align)
treeNJ <- NJ(dm)
fit = pml(treeNJ, data=phang.align)
```

```r
fitGTR <- update(fit, k=4, inv=0.2)
fitGTR <- optim.pml(fitGTR, model="GTR", optInv=TRUE, optGamma=TRUE,
                    rearrangement = "stochastic", control = pml.control(trace = 0))
detach("package:phangorn", unload=TRUE)

sp_tree <- fitGTR$tree
```

The package 'picante' is then used to calculate phylogenetic diversity.

```r
prunedTree <- prune.sample(asv_sp_rar_seqs,sp_tree)

sp_pd <- pd(asv_sp_rar_seqs, prunedTree, include.root = F)

sp_pd_id <- sp_pd %>%
  rownames_to_column(var = "ID")

meta_factors <- meta_sp_trimmed[,1:5] %>%
  rownames_to_column(var = "ID")

sp_pd_fac <- merge(meta_factors, sp_pd_id, by = "ID")
```

Fitting the best model to the data.

```r
shapiro.test(sp_pd_fac$PD)
```

```
##
##  Shapiro-Wilk normality test
##
## data:  sp_pd_fac$PD
## W = 0.98237, p-value = 0.1354
```

```r
sp_pd_lm <- aov(PD ~ GeographicRegion*Exposure*Zone*Depth, data = sp_pd_fac)

aSpPD_lm <- anova(sp_pd_lm)

sp_pd_lm1 <- update(sp_pd_lm, .~. -GeographicRegion:Exposure:Zone:Depth)
aSpPD_lm1 <-anova(sp_pd_lm1, test = "F")

sp_pd_lm2 <- update(sp_pd_lm1, .~. -Exposure:Zone:Depth)
aSpPD_lm2 <-anova(sp_pd_lm2, test = "F")

sp_pd_lm3 <- update(sp_pd_lm2, .~. -GeographicRegion:Zone:Depth)
aSpPD_lm3 <-anova(sp_pd_lm3, test = "F")

sp_pd_lm4 <- update(sp_pd_lm3, .~. -GeographicRegion:Exposure:Depth)
aSpPD_lm4 <-anova(sp_pd_lm4, test = "F")
```

Supementary Table S5

Analysis of variance table of Phylogenetic diversity (index)Faith's PD) indicating significant differences between the interaction factors geographic region, exposure, and zone.

```r
aSpPD_lm4
```

```
## Analysis of Variance Table
##
```

```
## Response: PD
##                                Df Sum Sq Mean Sq F value    Pr(>F)
## GeographicRegion                1 1292.2  1292.2 18.1090 4.609e-05 ***
## Exposure                        1    8.5     8.5  0.1187 0.7311321
## Zone                            1 2945.2  2945.2 41.2756 4.160e-09 ***
## Depth                           1  941.3   941.3 13.1923 0.0004401 ***
## GeographicRegion:Exposure       1 1025.0  1025.0 14.3655 0.0002538 ***
## GeographicRegion:Zone           1 4665.7  4665.7 65.3878 1.258e-12 ***
## Exposure:Zone                   1  761.6   761.6 10.6734 0.0014770 **
## GeographicRegion:Depth          1  354.1   354.1  4.9623 0.0280805 *
## Exposure:Depth                  1   44.9    44.9  0.6298 0.4292590
## Zone:Depth                      1  160.4   160.4  2.2476 0.1368837
## GeographicRegion:Exposure:Zone  1 1045.8  1045.8 14.6568 0.0002217 ***
## Residuals                     103 7349.6    71.4
## ---
## Signif. codes:  0 '***' 0.001 '**' 0.01 '*' 0.05 '.' 0.1 ' ' 1
```

```r
par(mfrow = c(2,2))
plot(sp_pd_lm4)
```

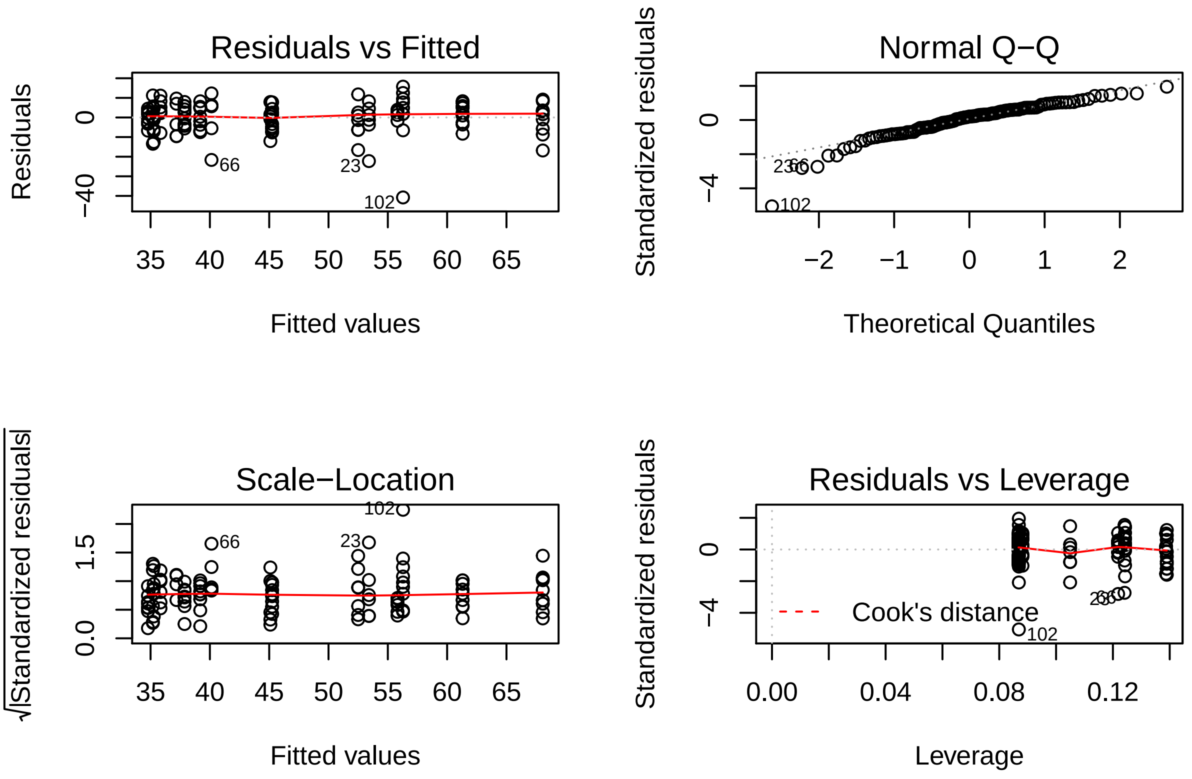


```r
par(mfrow = c(1,1))
```

Diagnostic plots

Diagnostic plots to visually evaluate the fit of the model.

Results of the pairwise comparison of Faith's PD across all interaction factors.

```r
TukeyHSD(sp_pd_lm4)
model.tables(sp_pd_lm4, "mean")
```

```r
ggplot(sp_pd_fac, aes(Zone, PD, colour = GeographicRegion, fill = Depth))+
  geom_boxplot(notch = T)+
  scale_fill_manual(values = c("gray90", "grey50"))+
  scale_colour_brewer(palette = "Dark2")+
  labs(y = "Faith's PD")+
  facet_grid(cols = vars(GeographicRegion), rows = vars(Exposure))+
  theme_bw()
```

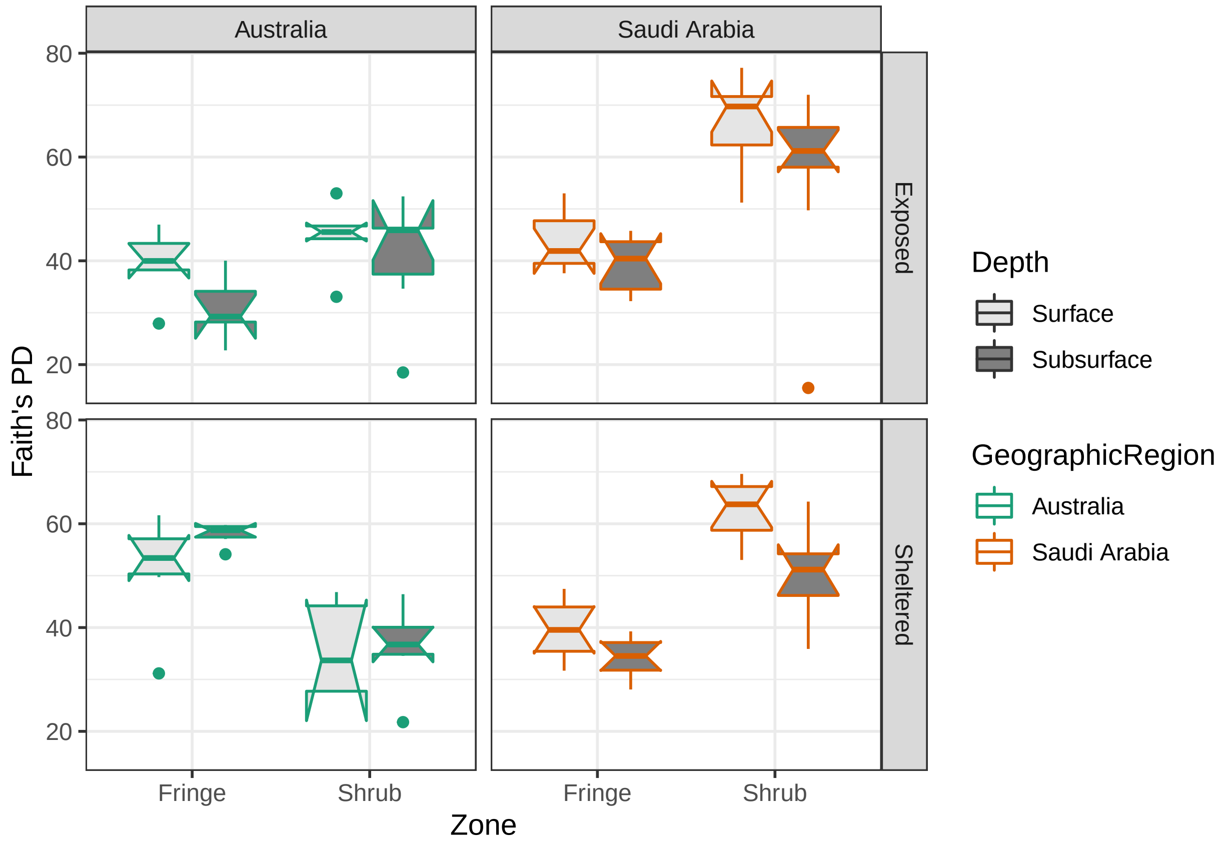

Figure 1D

Phylogenetic diversity (Faith's PD) across all experimental factors. The boxplots indicate the median with the interquartile range (IQR) between the 25th and 75th percentile, and the whiskers extend 1.5xIQR. Boxes were plotted with the notch (+/- 1.58xIQR/sqrt(n)) to display likely statistical significance if notches don't overlap.

5. Relative abundance

Phylum

Phyloseq lets us agglomerate the data to the taxonomic level we require and transform the counts to relative abundances using these commands.

```r
phyl <- tax_glom(sp_rar, "Phylum")
phyl_ra <- transform_sample_counts(phyl, function(x) x / sum(x)*100)
```

We display the names of the 13 most abundant Phyla and group the ramaining rare Phyla as "Others". To get a list of the most abundant phyla and their relative abundances across all samples we can use the following code:

Supplementary Table S6

Relative abundaces of phyla across all samples

```
phyl1 <- psmelt(phyl_ra)
phyl1$Phylum <- as.character(phyl1$Phylum)
phyl2 <- ddply(phyl1, .(Phylum), summarise, Abundance = mean(Abundance))
phyl3 <- arrange(phyl2, desc(Abundance))

other <- phyl3$Phylum[15:length(phyl3$Phylum)]
phyl1[phyl1$Phylum %in% other,]$Phylum <- "Other"
print(phyl3[1:20,], digits = 2)
```

```
##               Phylum Abundance
## 1     Proteobacteria     58.96
## 2      Bacteroidetes     17.44
## 3        Chloroflexi      8.06
## 4     Calditrichaeota     3.81
## 5        Nitrospirae      1.64
## 6      Cyanobacteria      1.59
## 7      Acidobacteria      1.42
## 8     Actinobacteria      1.29
## 9    Gemmatimonadetes     1.02
## 10       Spirochaetes     0.78
## 11     Planctomycetes     0.62
## 12       Dadabacteria     0.42
## 13       Zixibacteria     0.39
## 14 Epsilonbacteraeota     0.39
## 15   Halanaerobiaeota     0.33
## 16    Verrucomicrobia     0.26
## 17     Modulibacteria     0.19
## 18 Kiritimatiellaeota     0.18
## 19    Patescibacteria     0.16
## 20         Firmicutes     0.15
```

Class

Supplementary Table S7

Relative abundances of bacterial classes across all samples.

```
class <- tax_glom(sp_rar, "Class")
class_ra <- transform_sample_counts(class, function(x) x / sum(x)*100)
class1 <- psmelt(class_ra)

class2 <- ddply(class1, .(Class), summarise, Abundance = mean(Abundance))
class3 <- arrange(class2, desc(Abundance))
print(class3[1:20,])
```

```
##                      Class  Abundance
## 1      Deltaproteobacteria 31.5920718
## 2      Gammaproteobacteria 23.3393507
## 3              Bacteroidia 13.6548242
## 4             Anaerolineae  7.1244092
## 5      Alphaproteobacteria  4.4355564
## 6             Calditrichia  3.8438908
## 7             Rhodothermia  3.4149008
```

```
## 8          Oxyphotobacteria  1.5915434
## 9    Thermodesulfovibrionia  1.4156386
## 10           Acidimicrobiia  1.0997267
## 11          Dehalococcoidia  0.9535442
## 12      Thermoanaerobaculia  0.7755663
## 13 BD2-11_terrestrial_group  0.7588944
## 14             Spirochaetia  0.7317799
## 15           Ignavibacteria  0.5091801
## 16            Dadabacteriia  0.4231718
## 17          Campylobacteria  0.3929042
## 18            Halanaerobiia  0.3375212
## 19             Subgroup_21  0.3152048
## 20         Planctomycetacia  0.2808012
```

Order

```
ord <- tax_glom(sp_rar, "Order")
ord_ra <- transform_sample_counts(ord, function(x) x / sum(x)*100)
ord1 <- psmelt(ord_ra)

ord2 <- ddply(ord1, .(Order), summarise, Abundance = mean(Abundance))
ord3 <- arrange(ord2, desc(Abundance))
print(ord3[1:20,])
```

5.1 Relative abundance bar charts

Cosmetics for ggplot

This is to create an extra factor that combines Zone and Depth to fit both on the x-axis. We then rename and reorder the factor for visual representation

```
proteo_as_class <- tax_sp_rar
n <- rownames_to_column(proteo_as_class, "ASV")
setDT(n)
n[Phylum == "Proteobacteria", Phylum := Class ]
proteo_as_class <- column_to_rownames(n, "ASV")
proteo_as_class <- as.matrix(proteo_as_class)

ASV2 = otu_table(asv_sp_rar, taxa_are_rows = FALSE)
TAX2 = tax_table(proteo_as_class)
samples2 = sample_data(meta_sp_trimmed)
pr_as_cl <- phyloseq(ASV2, TAX2, samples2)


prot <- tax_glom(pr_as_cl, "Phylum")
prot_ra <- transform_sample_counts(prot, function(x) x / sum(x)*100)

prot1 <- psmelt(prot_ra)
prot1$Phylum <- as.character(prot1$Phylum)
prot2 <- ddply(prot1, .(Phylum), summarise, Abundance = mean(Abundance))
prot3 <- arrange(prot2, desc(Abundance))

other <- prot3$Phylum[15:length(prot3$Phylum)]
prot1[prot1$Phylum %in% other,]$Phylum <- "Other"
```

```
w1 <- prot1
w2 <- ddply(w1, .(GeographicRegion, Exposure, Zone, Depth, Phylum),
            summarise, Abundance = mean(Abundance))
w3 <- unite(w2, ZoneDepth, Zone, Depth, remove = F)
w3$ZoneDepth <- as.factor(w3$ZoneDepth)
levels(w3$ZoneDepth) <- c("Fringe Subsurface", "Fringe Surface",
                          "Shrub Subsurface", "Shrub Surface")
w3$ZoneDepth <- factor(w3$ZoneDepth, levels=c("Fringe Surface","Fringe Subsurface",
                                              "Shrub Surface","Shrub Subsurface"))

w3$Phylum <- factor(w3$Phylum, levels =
                      c("Acidobacteria","Actinobacteria","Bacteroidetes",
                        "Calditrichaeota","Chloroflexi", "Cyanobacteria",
                        "Dadabacteria", "Gemmatimonadetes","Nitrospirae",
                        "Planctomycetes","Alphaproteobacteria","Deltaproteobacteria",
                        "Gammaproteobacteria", "Spirochaetes", "Other"))
```

Choosing a classic colour palette "Paired", with 12 colors, that can be extended via the colorRampPalette command. We assign the names of our "Phylum" factor to those colours and store them in an objet to be used in the plot command.We crate the graph using ggplot2.

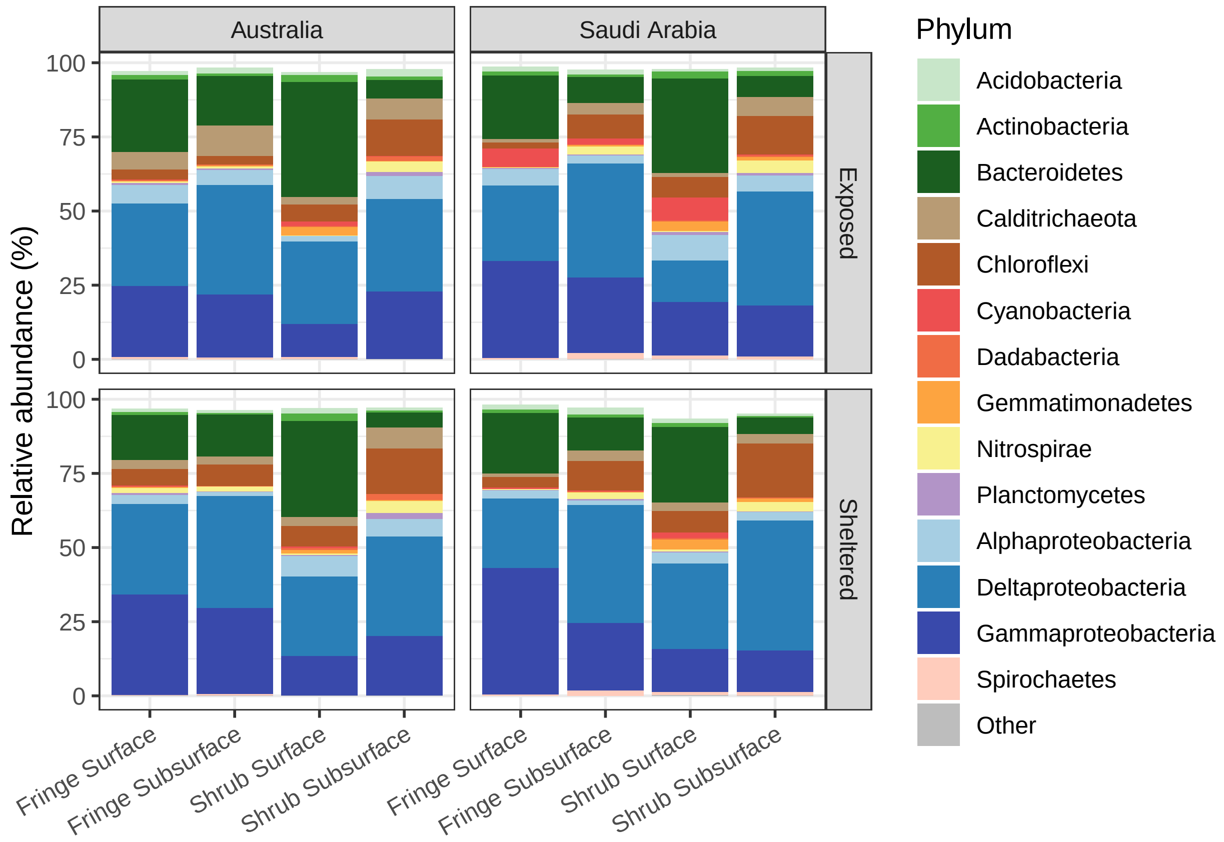

Figure 2A

Relative abundance of phyla across all factors.

5.2 Variation of five most abundant phyla (90 %) across factors

This chunk creates bar plots of the most abundant phyla that shows how their relative abundance changes across the different factors.

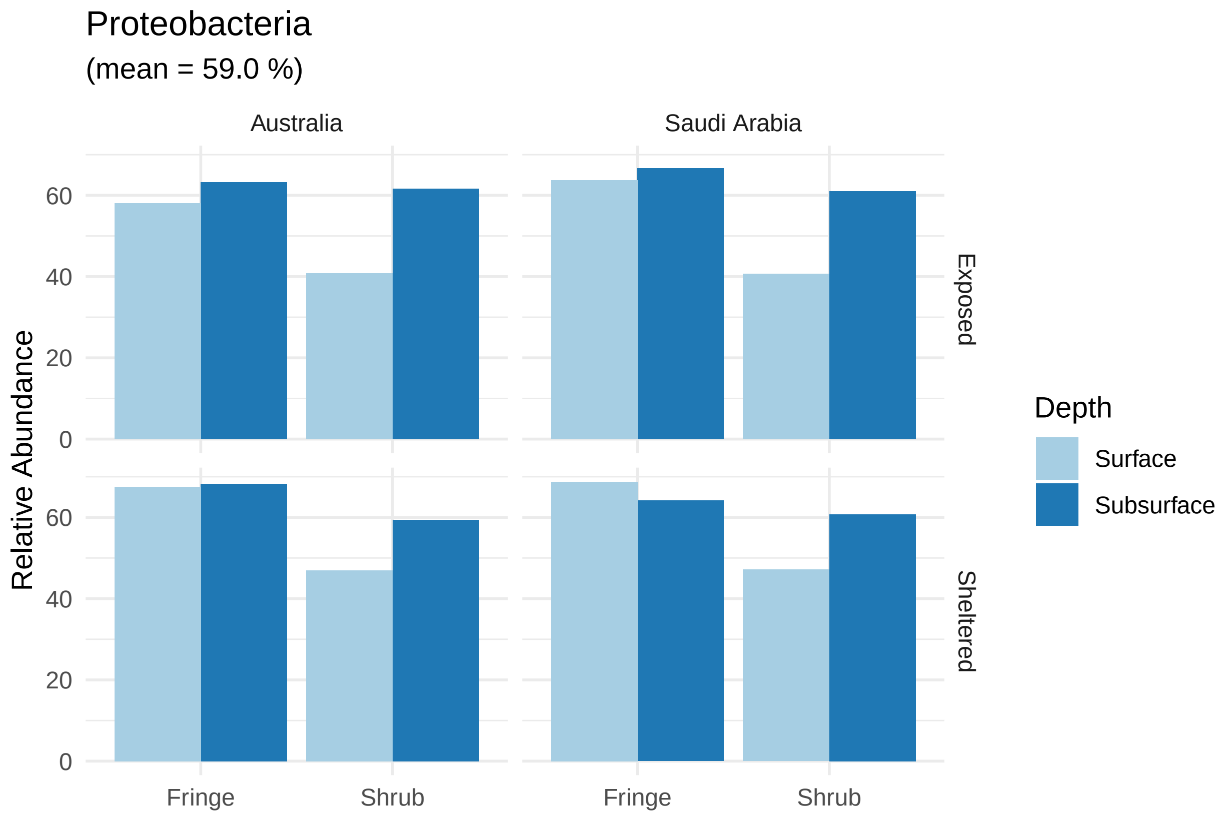

Supplementary Figure S3

Relative abundance of Proteobacteria across all factors. The mean given above represents the relative abundance across all samples.

```r
### Bacteriodetes
bact <- subset_taxa(phyl_ra, Phylum == "Bacteroidetes")
bact <- psmelt(bact)
bact1 <- ddply(bact, .(GeographicRegion, Exposure, Zone, Depth, Phylum),
               summarise, Abundance=mean(Abundance))

ph2 <- ggplot(bact1, aes(Zone, Abundance, fill = Depth))+
  geom_bar(stat = "identity", position = "dodge")+
  scale_fill_brewer(palette = "Paired")+
  facet_grid(Exposure ~ GeographicRegion)+
  labs(title = "Bacteroidetes", subtitle = "mean = 17.4 %",
       x = "", y = "Relative Abundance")+
  theme_minimal()
ph2
```

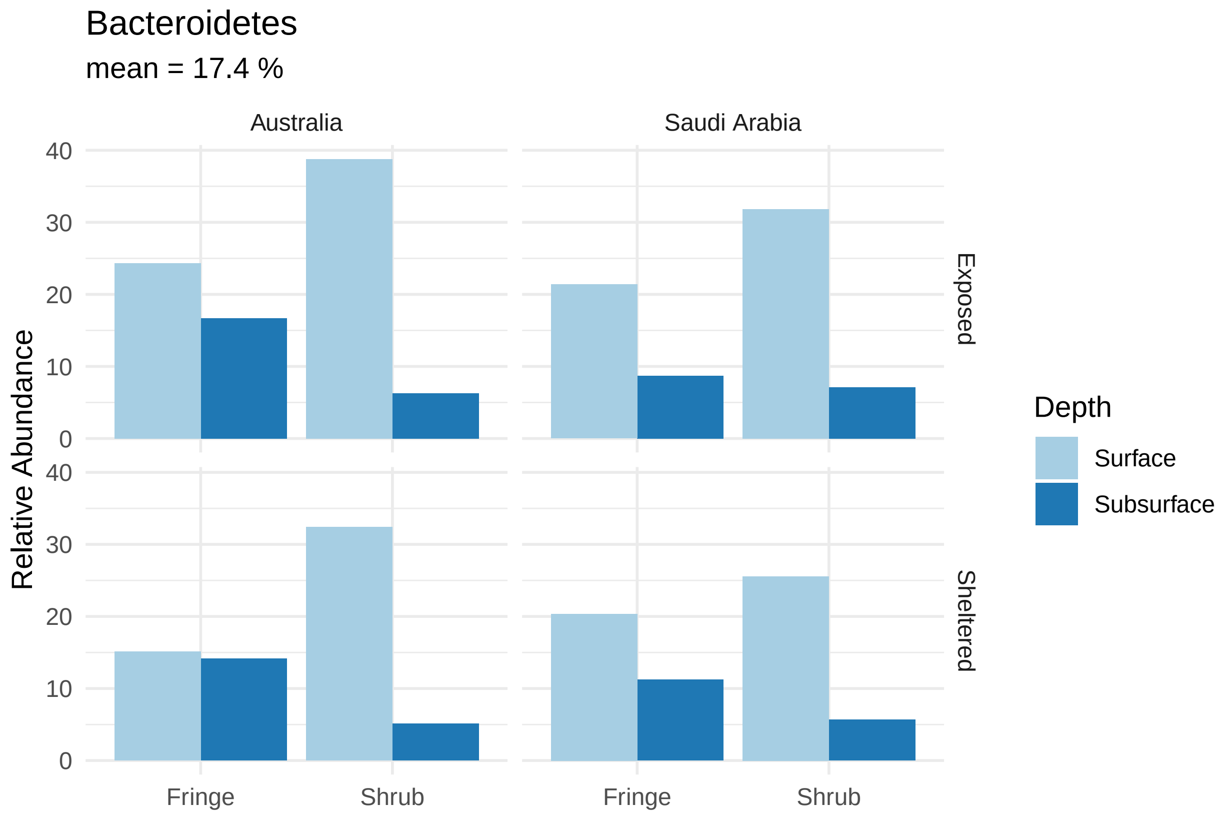

Supplementary Figure S4

Relative abundance of Bacteroidetes across all factors. The mean given above represents the relative abundance across all samples.

```
### Chloroflexi
chlo <- subset_taxa(phyl_ra, Phylum == "Chloroflexi")
chlo <- psmelt(chlo)
chlo1 <- ddply(chlo, .(GeographicRegion, Exposure, Zone, Depth, Phylum),
               summarise, Abundance=mean(Abundance))


ph3 <- ggplot(chlo1, aes(Zone, Abundance, fill = Depth))+
  geom_bar(stat = "identity", position = "dodge")+
  scale_fill_brewer(palette = "Paired")+
  facet_grid(Exposure ~ GeographicRegion)+
  labs(title = "Chloroflexi", subtitle = "mean = 8.1 %",
       x = "", y = "Relative Abundance")+
  theme_minimal()
ph3
```

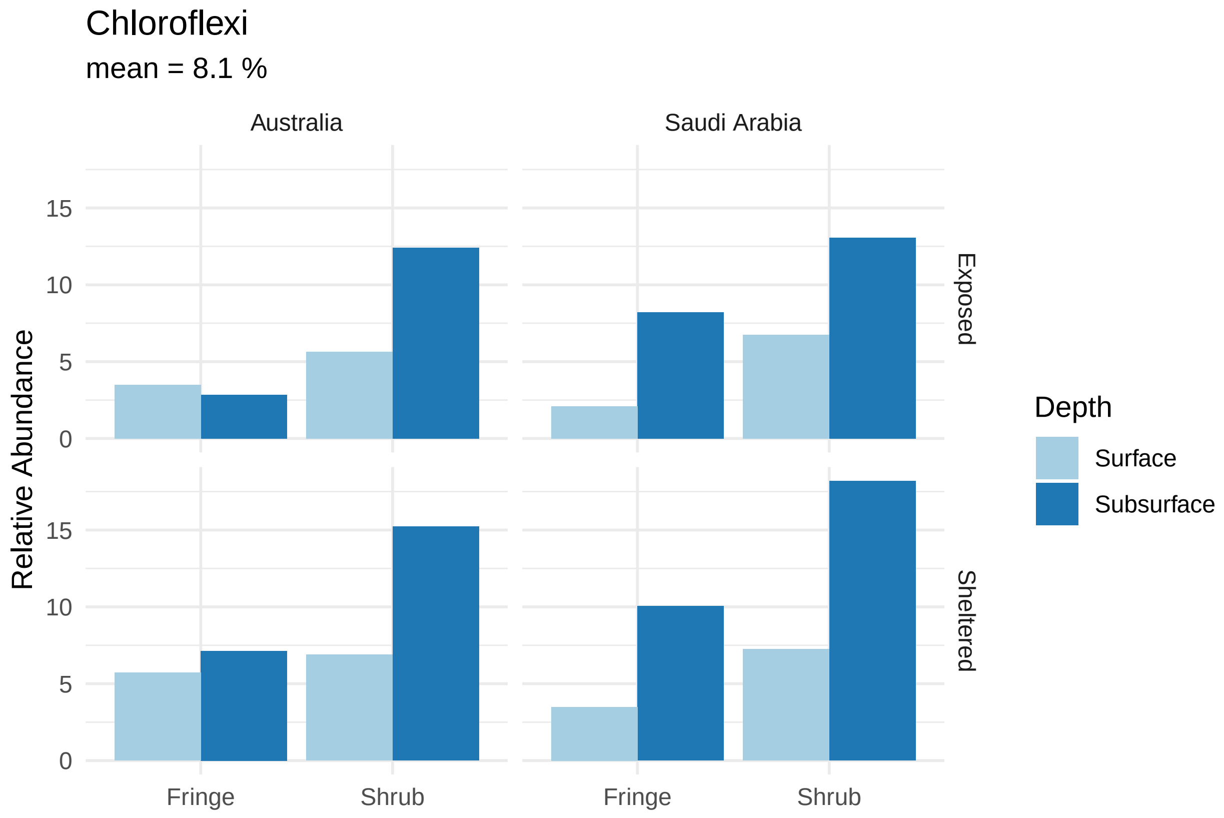

Supplementary Figure S5

Relative abundance of Chloroflexi across all factors. The mean given above represents the relative abundance across all samples.

```
### Calditrichaeota
cald <- subset_taxa(phyl_ra, Phylum == "Calditrichaeota")
cald <- psmelt(cald)
cald1 <- ddply(cald, .(GeographicRegion, Exposure, Zone, Depth, Phylum),
               summarise, Abundance=mean(Abundance))

ph4 <- ggplot(cald1, aes(Zone, Abundance, fill = Depth))+
  geom_bar(stat = "identity", position = "dodge")+
  scale_fill_brewer(palette = "Paired")+
  facet_grid(Exposure ~ GeographicRegion)+
  labs(title = "Calditrichaeota", subtitle = "mean = 3.8 %",
       x = "", y = "Relative Abundance")+
  theme_minimal()
ph4
```

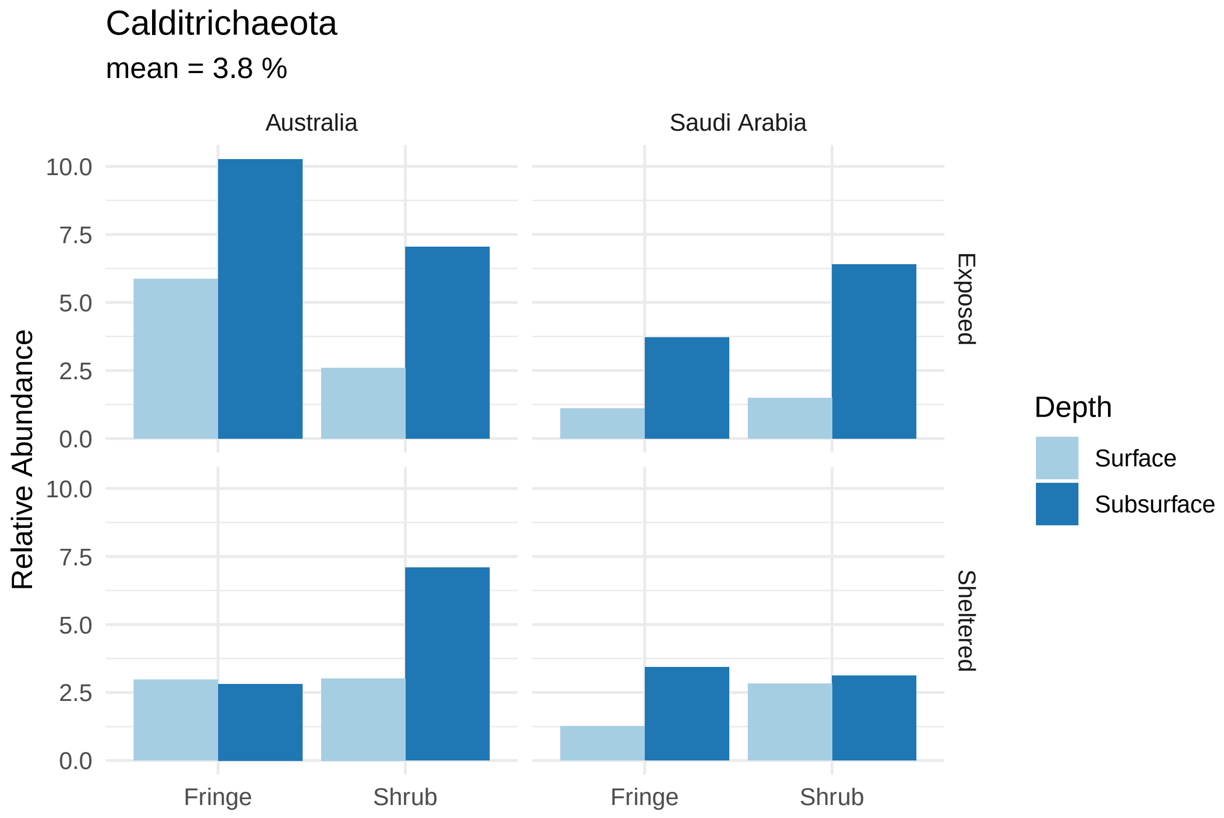

Supplementary Figure S6

Relative abundance of Calditrichaeota across all factors. The mean given above represents the relative abundance across all samples.

```
### Nitrospirae
nit <- subset_taxa(phyl_ra, Phylum == "Nitrospirae")
nit <- psmelt(nit)
nit1 <- ddply(nit, .(GeographicRegion, Exposure, Zone, Depth, Phylum),
              summarise, Abundance=mean(Abundance))

ph5 <- ggplot(nit1, aes(Zone, Abundance, fill = Depth))+
  geom_bar(stat = "identity", position = "dodge")+
  scale_fill_brewer(palette = "Paired")+
  facet_grid(Exposure ~ GeographicRegion)+
  labs(title = "Nitrospirae", subtitle = "mean = 1.6 %",
       x = "", y = "Relative Abundance")+
  theme_minimal()
ph5
```

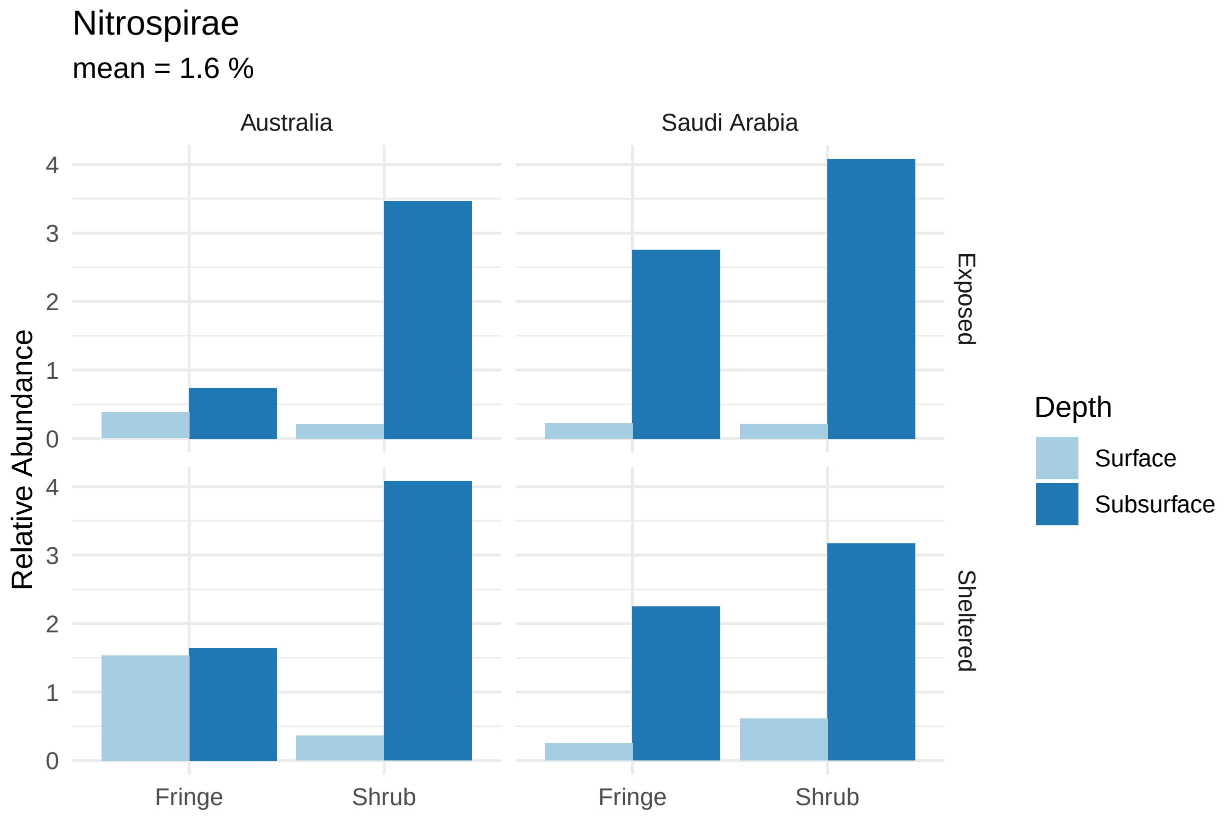

Supplementary Figure S7

Relative abundance of Nitrospirae across all factors. The mean given above represents the relative abundance across all samples.

```
### Cyanobacteria
cyano <- subset_taxa(phyl_ra, Phylum == "Cyanobacteria")
cyano <- psmelt(cyano)
cyano1 <- ddply(cyano, .(GeographicRegion, Exposure, Zone, Depth, Phylum),
                summarise, Abundance=mean(Abundance))

ph6 <- ggplot(cyano1, aes(Zone, Abundance, fill = Depth))+
  geom_bar(stat = "identity", position = "dodge")+
  scale_fill_brewer(palette = "Paired")+
  facet_grid(Exposure ~ GeographicRegion)+
  labs(title = "Cyanobacteria", subtitle = "mean = 1.6 %",
       x = "", y = "Relative Abundance")+
  theme_minimal()
ph6
```

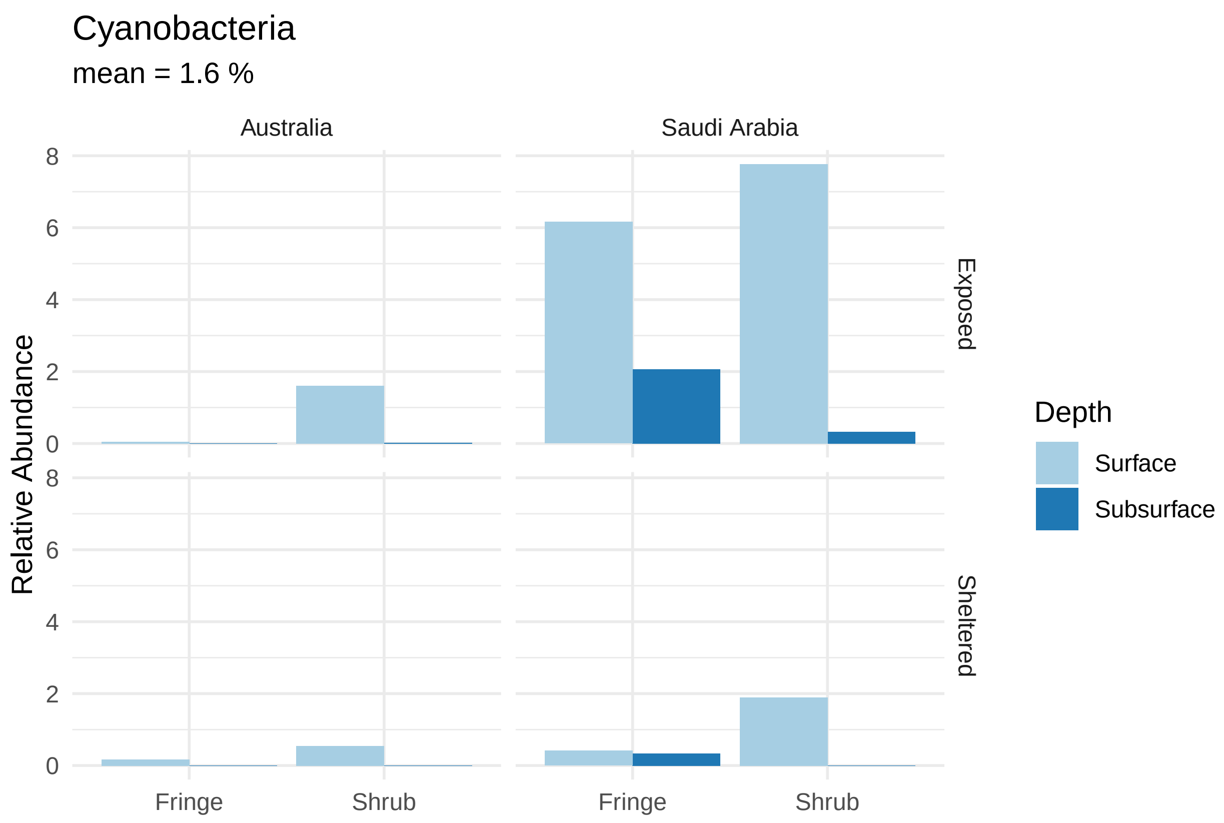

Supplementary Figure S8

Relative abundance of Cyanobacteria across all factors. The mean given above represents the relative abundance across all samples.

6 Beta diversity

We visualise beta diversity using a distance based ordination method and test the differences between factors using PERMANOVA.

The quality of the representation of the ordination method in 2 dimensions can be checked and visualised with a "Shepard" diagram. It represents the "distance in the 2 dimensions of an ordination", plotted against the original distance in the k dimensional space "true bray-curtis distance". Here, we compare the quality of the representation in 2 dimensions of a NMDS and a MDS (PCoA) based on the Bray-Curtis distance.

For that we use the "metaMDS" command for NMDS, and the "cmdscale" with the "vegdist" command for PCoA, to calcualte the distance metrices based on Bray-Curtis method

```
nmds <- metaMDS(asv_sp_rar, distance = "bray", k = 2, trymax = 50)
mds <- cmdscale(vegdist(asv_sp_rar, method = "bray"), k = 2, eig = T, add = T )
```

6.0.1 NMDS vs PCoA

We then plot the distance in the reduced space (between the two axes) against the true bray-curtis distance

```
par(mfrow = c(1,2), mar = c(3.5,3.5,3,1), mgp = c(2, 0.6, 0), cex = 0.8, las = 1)
spear <- round(cor(vegdist(asv_sp_rar, method = "bray"), dist(nmds$points),
                   method = "spearman"),3)
plot(vegdist(asv_sp_rar, method = "bray"), dist(nmds$points),
```

```
     main = "Shepard diagram of NMDS",
     xlab = "True Bray-Curtis distance", ylab = "Distance in the reduced space")
mtext(line = 0.1, text = paste0("Spearman correlation = ", spear), cex = 0.7)

spear <- round(cor(vegdist(asv_sp_rar, method = "bray"), dist(mds$points),
                   method = "spearman"),3)
plot(vegdist(asv_sp_rar, method = "bray"), dist(mds$points),
     main = "Shepard diagram of PCoA",
     xlab = "True Bray-Curtis distance", ylab = "Distance in the reduced space")
mtext(line = 0.1, text = paste0("Spearman correlation = ", spear), cex = 0.7)
```

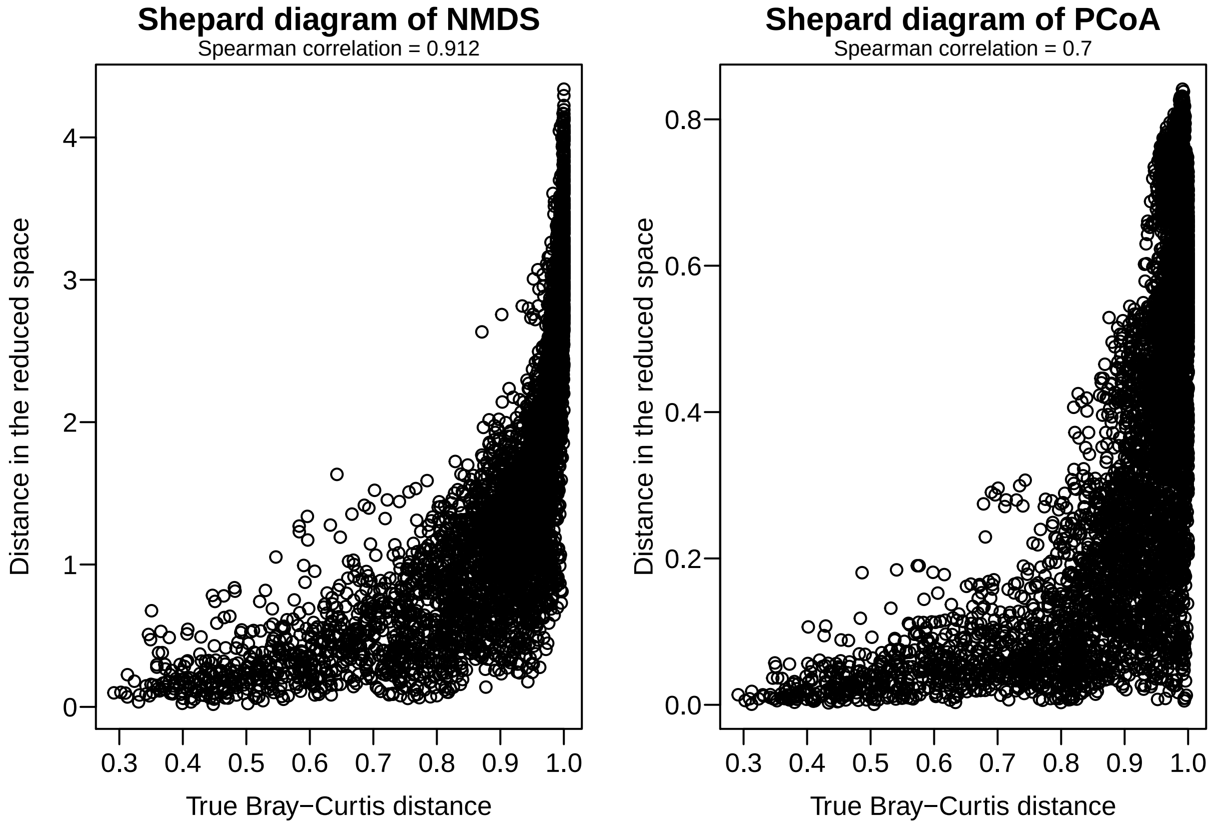

Diagnostic plot

This shepard diagram with with spearman corellation helps to investigate the 'goodness of fit' of the ordination method, hence, we choose NMDS to be the preferred method, as the correlation is stronger (R^2 = 0.91) compared to that of the PCoA (R^2 = 0.69).

6.1 Non Metric Multidimensional Scaling (NMDS)

We use the metaMDS call from above.

Check Stress:

```
## [1] 0.179292
```

We create a data frame by joining the coordinates generated by the "metaMDS" command with the required meta data.

```
NMDS <- data.frame(x = nmds$point[,1], y = nmds$point[,2],
                   GeographicRegion = as.factor(meta_sp_trimmed[,1]),
```

```
              Exposure = as.factor(meta_sp_trimmed[,3]),
              Zone = as.factor(meta_sp_trimmed[,4]),
              Depth = as.factor(meta_sp_trimmed[,5]))
```

6.1.1 NMDS plot at ASV level across all factors

And plot the results with ggplot2:

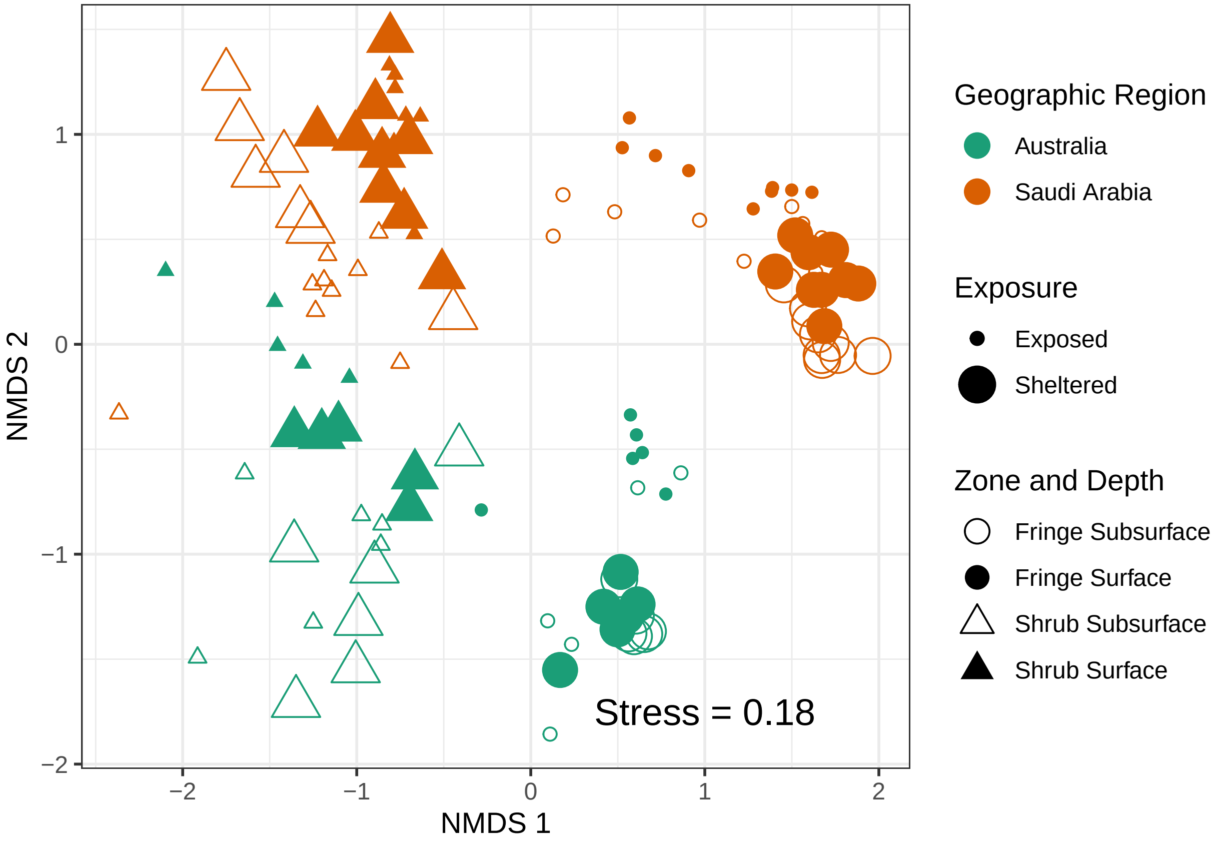

Figure 2C

NMDS plot of the community matrix across all experimental factors at ASV level.

A quick test of PCoA Eigenvalues in phyloseq can give a rough estimate on the amount of variation that can be explained by the first two dimensions. It is important to note, that these two methods work differently and that NMDS cannot give a number to the variation it is able to capture. The values given from the PCoA plot can however be informative as to how well the data is explained.

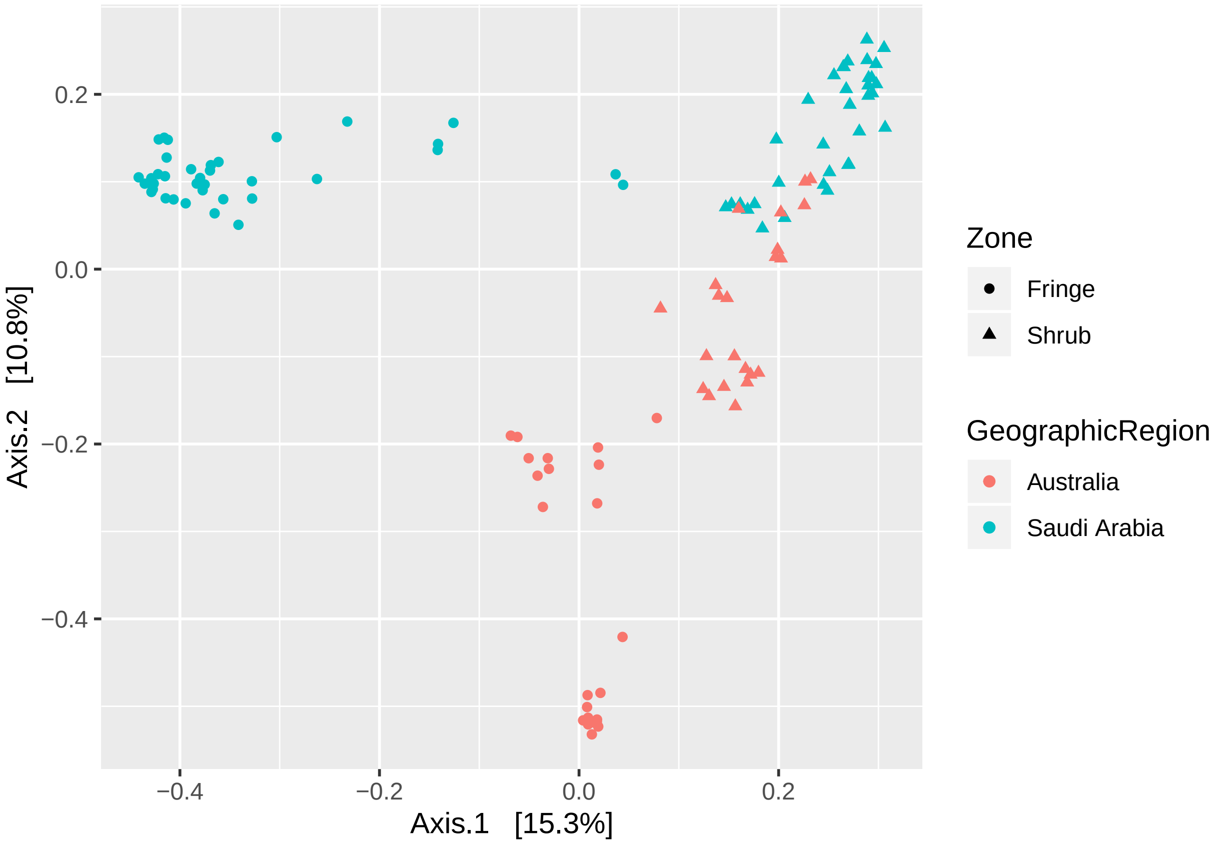

Diagnostic plot

The first axis, which the data mainly by Zone, explains 15,3 % of the variation, and the second axis, along which the Geographic Regions separate, explains 10.8 %. A total of 26.1 % of variation is explained by the two axes of the PCoA.

6.1.2 NMD at genus level

```
nmds_gen <- metaMDS(as.data.frame(otu_table(gen)), distance = "bray", k = 2, trymax = 50)
```

Check stress.

```
print(nmds_gen$stress)
```

```
## [1] 0.1850955
```

```
NMDS_gen <- data.frame(x = nmds_gen$point[,1], y = nmds_gen$point[,2],
                GeographicRegion = as.factor(meta_sp_trimmed[,1]),
                Exposure = as.factor(meta_sp_trimmed[,3]),
                Zone = as.factor(meta_sp_trimmed[,4]),
                Depth = as.factor(meta_sp_trimmed[,5]))
NMDS_gen <- unite(NMDS_gen, shape_fill, Zone, Depth, remove = F)

ggplot(NMDS_gen, aes(x, y, colour = GeographicRegion,
                     shape = shape_fill, size = Exposure))+
  geom_point()+
  scale_colour_brewer(palette = "Dark2")+
  scale_shape_manual(values = c(1, 16, 2, 17),
                     name = "Zone and Depth",
```

```r
                    labels = c("Fringe Subsurface",
                               "Fringe Surface",
                               "Shrub Subsurface",
                               "Shrub Surface"))+
guides(colour = guide_legend(title = "Geographic Region", order = 1, override.aes = list(size = 4)),
       size = guide_legend(order = 2),
       shape = guide_legend(order = 3, override.aes = list(size = 4)))+
labs(x = "NMDS 1", y = "NMDS 2")+
theme_bw()+
annotate("text", x = .5, y = -1.25, label = "Stress = 0.19", size = 5)
```

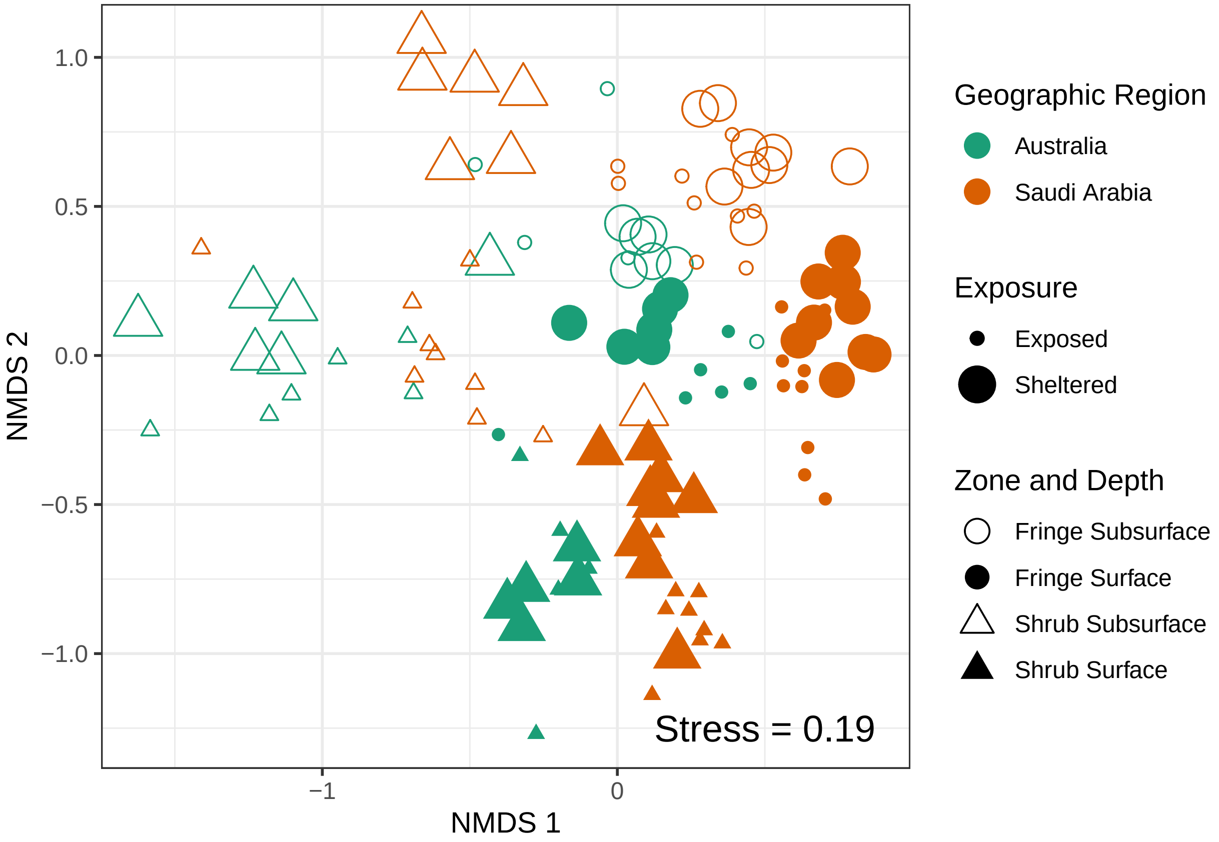

Figure 2D

NMDS plot of the community matrix across all experimental factors at genus level.

6.2 PERMANOVA

In preparation for the hypothesis test, the abundance data will be log+1 transformed and converted into a distance matrix using bray-curtis distance. We then test for the homogeneity of the multivariate dispersion (an assumption for the PERMANOVA).

```r
sp_log <- transform_sample_counts(sp_rar, function(x) log(1 + x) )

sp_log_bray <- phyloseq::distance(sp_log, method = "bray")

log_disp_c <- betadisper(sp_log_bray, meta_sp_trimmed$GeographicRegion)
log_disp_e <- betadisper(sp_log_bray, meta_sp_trimmed$Exposure)
```

```r
log_disp_z <- betadisper(sp_log_bray, meta_sp_trimmed$Zone)
log_disp_d <- betadisper(sp_log_bray, meta_sp_trimmed$Depth)

anova(log_disp_c)
```

```
## Analysis of Variance Table
##
## Response: Distances
##             Df   Sum Sq   Mean Sq F value Pr(>F)
## Groups       1 0.000736 0.0007356  0.4318 0.5125
## Residuals 113 0.192513 0.0017036
```

```r
anova(log_disp_e)
```

```
## Analysis of Variance Table
##
## Response: Distances
##             Df   Sum Sq    Mean Sq F value Pr(>F)
## Groups       1 0.000951 0.00095107  0.9301 0.3369
## Residuals 113 0.115547 0.00102254
```

```r
anova(log_disp_z)
```

```
## Analysis of Variance Table
##
## Response: Distances
##             Df  Sum Sq   Mean Sq F value Pr(>F)
## Groups       1 0.00079 0.0007863  0.1878 0.6656
## Residuals 113 0.47319 0.0041876
```

```r
anova(log_disp_d)
```

```
## Analysis of Variance Table
##
## Response: Distances
##             Df   Sum Sq   Mean Sq F value Pr(>F)
## Groups       1 0.002931 0.0029310  2.3126 0.1311
## Residuals 113 0.143220 0.0012674
```

The p-values indicate an even dispersion of the variance between all factors which means we can proceed with the analysis.

The adonis2 command performs a PERMANOVA comparison across all factors on the distance matrix.

```r
ad1 <- adonis2(sp_log_bray~GeographicRegion*Exposure*Zone*Depth,
               meta_sp_trimmed, permutations = 999,
               strata = NULL, contr.unordered = "contr.sum",
               contr.ordered = "contr.poly")
```

Suppementary Table S8

Summary table showing the results of the PERMANOVA on the abundance table at ASV level with significant differences between the interaction factors geographic region, exposure, zone, and depth.

```r
print(ad1)
```

```
## Permutation test for adonis under reduced model
## Terms added sequentially (first to last)
```

```
## Permutation: free
## Number of permutations: 999
##
## adonis2(formula = sp_log_bray ~ GeographicRegion * Exposure * Zone * Depth, data = meta_sp_trimmed, p
##                                         Df SumOfSqs      R2       F Pr(>F)
## GeographicRegion                         1    5.034 0.10645 35.1629  0.001
## Exposure                                 1    2.449 0.05179 17.1082  0.001
## Zone                                     1    6.983 0.14766 48.7758  0.001
## Depth                                    1    2.688 0.05684 18.7761  0.001
## GeographicRegion:Exposure                1    2.276 0.04812 15.8952  0.001
## GeographicRegion:Zone                    1    3.735 0.07898 26.0885  0.001
## Exposure:Zone                            1    1.965 0.04154 13.7234  0.001
## GeographicRegion:Depth                   1    1.185 0.02506  8.2771  0.001
## Exposure:Depth                           1    0.568 0.01201  3.9659  0.001
## Zone:Depth                               1    1.887 0.03990 13.1788  0.001
## GeographicRegion:Exposure:Zone           1    1.983 0.04194 13.8539  0.001
## GeographicRegion:Exposure:Depth          1    0.539 0.01140  3.7670  0.001
## GeographicRegion:Zone:Depth              1    0.869 0.01837  6.0668  0.001
## Exposure:Zone:Depth                      1    0.478 0.01010  3.3359  0.001
## GeographicRegion:Exposure:Zone:Depth     1    0.480 0.01016  3.3556  0.001
## Residual                                99   14.174 0.29970
## Total                                  114   47.293 1.00000
##
## GeographicRegion                       ***
## Exposure                               ***
## Zone                                   ***
## Depth                                  ***
## GeographicRegion:Exposure              ***
## GeographicRegion:Zone                  ***
## Exposure:Zone                          ***
## GeographicRegion:Depth                 ***
## Exposure:Depth                         ***
## Zone:Depth                             ***
## GeographicRegion:Exposure:Zone         ***
## GeographicRegion:Exposure:Depth        ***
## GeographicRegion:Zone:Depth            ***
## Exposure:Zone:Depth                    ***
## GeographicRegion:Exposure:Zone:Depth ***
## Residual
## Total
## ---
## Signif. codes:  0 '***' 0.001 '**' 0.01 '*' 0.05 '.' 0.1 ' ' 1
```

```
AllFactors <- unite(meta_sp_trimmed, allFactors,
                    GeographicRegion, Exposure, Zone, Depth, remove = F)

pwad <- pairwise.adonis2(sp_log_bray ~ allFactors, data = AllFactors)
```

6.2.1 Comparison between geographic regions

For the pairwise comprison of the PERMANOVA results, the reasonable combinations were extracted and ordered by factor of comparison

Supplementary Table S9

Pairwise comparisons of the significant four-way interactions between ecologically relevant combinations of the factors geographic region, exposure, zone, and depth.

```
pwad$`Australia_Sheltered_Shrub_Subsurface_vs_Saudi Arabia_Sheltered_Shrub_Subsurface`
```

```
## Permutation: free
## Number of permutations: 999
##
## Terms added sequentially (first to last)
##
##             Df SumsOfSqs MeanSqs F.Model     R2 Pr(>F)
## allFactors  1    2.1646 2.16460  16.147 0.5948  0.001 ***
## Residuals  11    1.4746 0.13405         0.4052
## Total      12    3.6392                 1.0000
## ---
## Signif. codes:  0 '***' 0.001 '**' 0.01 '*' 0.05 '.' 0.1 ' ' 1
```

```
pwad$`Australia_Sheltered_Shrub_Subsurface_vs_Saudi Arabia_Sheltered_Fringe_Subsurface`
```

```
## Permutation: free
## Number of permutations: 999
##
## Terms added sequentially (first to last)
##
##             Df SumsOfSqs MeanSqs F.Model      R2 Pr(>F)
## allFactors  1    2.7549  2.7549  21.952 0.62806  0.001 ***
## Residuals  13    1.6315  0.1255         0.37194
## Total      14    4.3864                 1.00000
## ---
## Signif. codes:  0 '***' 0.001 '**' 0.01 '*' 0.05 '.' 0.1 ' ' 1
```

```
pwad$`Australia_Sheltered_Fringe_Subsurface_vs_Saudi Arabia_Sheltered_Fringe_Subsurface`
```

```
## Permutation: free
## Number of permutations: 999
##
## Terms added sequentially (first to last)
##
##             Df SumsOfSqs MeanSqs F.Model      R2 Pr(>F)
## allFactors  1    2.6416 2.64157  24.732 0.65547  0.002 **
## Residuals  13    1.3885 0.10681         0.34453
## Total      14    4.0300                 1.00000
## ---
## Signif. codes:  0 '***' 0.001 '**' 0.01 '*' 0.05 '.' 0.1 ' ' 1
```

```
pwad$`Australia_Exposed_Shrub_Subsurface_vs_Saudi Arabia_Exposed_Shrub_Subsurface`
```

```
## Permutation: free
## Number of permutations: 999
##
## Terms added sequentially (first to last)
##
##             Df SumsOfSqs MeanSqs F.Model      R2 Pr(>F)
## allFactors  1    1.5214 1.52143  9.8531 0.43115  0.001 ***
## Residuals  13    2.0074 0.15441         0.56885
```

```
## Total      14    3.5288                 1.00000
## ---
## Signif. codes:  0 '***' 0.001 '**' 0.01 '*' 0.05 '.' 0.1 ' ' 1
```

```
pwad$`Australia_Exposed_Fringe_Subsurface_vs_Saudi Arabia_Exposed_Fringe_Subsurface`
```

```
## Permutation: free
## Number of permutations: 999
##
## Terms added sequentially (first to last)
##
##            Df SumsOfSqs MeanSqs F.Model      R2 Pr(>F)
## allFactors  1    1.7990 1.79905  8.7777 0.42246  0.001 ***
## Residuals  12    2.4595 0.20496         0.57754
## Total      13    4.2585                 1.00000
## ---
## Signif. codes:  0 '***' 0.001 '**' 0.01 '*' 0.05 '.' 0.1 ' ' 1
```

```
pwad$`Australia_Sheltered_Shrub_Surface_vs_Saudi Arabia_Sheltered_Shrub_Surface`
```

```
## Permutation: free
## Number of permutations: 999
##
## Terms added sequentially (first to last)
##
##            Df SumsOfSqs MeanSqs F.Model      R2 Pr(>F)
## allFactors  1    1.6234 1.62335  11.045 0.47928  0.002 **
## Residuals  12    1.7637 0.14698         0.52072
## Total      13    3.3871                 1.00000
## ---
## Signif. codes:  0 '***' 0.001 '**' 0.01 '*' 0.05 '.' 0.1 ' ' 1
```

```
pwad$`Australia_Sheltered_Fringe_Surface_vs_Saudi Arabia_Sheltered_Fringe_Surface`
```

```
## Permutation: free
## Number of permutations: 999
##
## Terms added sequentially (first to last)
##
##            Df SumsOfSqs MeanSqs F.Model      R2 Pr(>F)
## allFactors  1    2.5270 2.52702  19.991 0.60596  0.001 ***
## Residuals  13    1.6433 0.12641         0.39404
## Total      14    4.1703                 1.00000
## ---
## Signif. codes:  0 '***' 0.001 '**' 0.01 '*' 0.05 '.' 0.1 ' ' 1
```

```
pwad$`Australia_Exposed_Shrub_Surface_vs_Saudi Arabia_Exposed_Shrub_Surface`
```

```
## Permutation: free
## Number of permutations: 999
##
## Terms added sequentially (first to last)
##
##            Df SumsOfSqs MeanSqs F.Model      R2 Pr(>F)
## allFactors  1    1.5483 1.54827  12.469 0.50959  0.001 ***
## Residuals  12    1.4900 0.12417         0.49041
## Total      13    3.0383                 1.00000
```

```
## ---
## Signif. codes:  0 '***' 0.001 '**' 0.01 '*' 0.05 '.' 0.1 ' ' 1
```

```
pwad$`Australia_Exposed_Fringe_Surface_vs_Saudi Arabia_Exposed_Fringe_Surface`
```

```
## Permutation: free
## Number of permutations: 999
##
## Terms added sequentially (first to last)
##
##            Df SumsOfSqs MeanSqs F.Model      R2 Pr(>F)
## allFactors  1    2.1977 2.19766  14.674 0.53025  0.001 ***
## Residuals  13    1.9469 0.14976         0.46975
## Total      14    4.1446                 1.00000
## ---
## Signif. codes:  0 '***' 0.001 '**' 0.01 '*' 0.05 '.' 0.1 ' ' 1
```

6.2.2 Comparison within Australia

```
pwad$Australia_Sheltered_Shrub_Subsurface_vs_Australia_Sheltered_Fringe_Subsurface
```

```
## Permutation: free
## Number of permutations: 999
##
## Terms added sequentially (first to last)
##
##            Df SumsOfSqs MeanSqs F.Model      R2 Pr(>F)
## allFactors  1   2.20959 2.20959  29.146 0.74455  0.003 **
## Residuals  10   0.75811 0.07581         0.25545
## Total      11   2.96771                 1.00000
## ---
## Signif. codes:  0 '***' 0.001 '**' 0.01 '*' 0.05 '.' 0.1 ' ' 1
```

```
pwad$Australia_Sheltered_Shrub_Subsurface_vs_Australia_Exposed_Shrub_Subsurface
```

```
## Permutation: free
## Number of permutations: 999
##
## Terms added sequentially (first to last)
##
##            Df SumsOfSqs MeanSqs F.Model      R2 Pr(>F)
## allFactors  1    1.2542 1.25424  9.5436 0.48832  0.001 ***
## Residuals  10    1.3142 0.13142         0.51168
## Total      11    2.5685                 1.00000
## ---
## Signif. codes:  0 '***' 0.001 '**' 0.01 '*' 0.05 '.' 0.1 ' ' 1
```

```
pwad$Australia_Sheltered_Shrub_Subsurface_vs_Australia_Sheltered_Shrub_Surface
```

```
## Permutation: free
## Number of permutations: 999
##
## Terms added sequentially (first to last)
##
##            Df SumsOfSqs MeanSqs F.Model     R2 Pr(>F)
## allFactors  1    1.4442 1.44416  12.839 0.5879  0.004 **
## Residuals   9    1.0123 0.11248         0.4121
```

```
## Total      10    2.4565                 1.0000
## ---
## Signif. codes:  0 '***' 0.001 '**' 0.01 '*' 0.05 '.' 0.1 ' ' 1
```

```
pwad$Australia_Sheltered_Fringe_Subsurface_vs_Australia_Exposed_Fringe_Subsurface
```

```
## Permutation: free
## Number of permutations: 999
##
## Terms added sequentially (first to last)
##
##            Df SumsOfSqs MeanSqs F.Model      R2 Pr(>F)
## allFactors  1   1.67393  1.6739  16.976 0.65353  0.001 ***
## Residuals   9   0.88743  0.0986         0.34647
## Total      10   2.56136                 1.00000
## ---
## Signif. codes:  0 '***' 0.001 '**' 0.01 '*' 0.05 '.' 0.1 ' ' 1
```

```
pwad$Australia_Sheltered_Fringe_Subsurface_vs_Australia_Sheltered_Fringe_Surface
```

```
## Permutation: free
## Number of permutations: 999
##
## Terms added sequentially (first to last)
##
##            Df SumsOfSqs  MeanSqs F.Model      R2 Pr(>F)
## allFactors  1   0.20313 0.203131  2.4152 0.19454  0.004 **
## Residuals  10   0.84104 0.084104         0.80546
## Total      11   1.04417                  1.00000
## ---
## Signif. codes:  0 '***' 0.001 '**' 0.01 '*' 0.05 '.' 0.1 ' ' 1
```

```
pwad$Australia_Exposed_Shrub_Subsurface_vs_Australia_Exposed_Fringe_Subsurface
```

```
## Permutation: free
## Number of permutations: 999
##
## Terms added sequentially (first to last)
##
##            Df SumsOfSqs MeanSqs F.Model     R2 Pr(>F)
## allFactors  1    1.5544 1.55444  9.6914 0.5185  0.004 **
## Residuals   9    1.4436 0.16039         0.4815
## Total      10    2.9980                 1.0000
## ---
## Signif. codes:  0 '***' 0.001 '**' 0.01 '*' 0.05 '.' 0.1 ' ' 1
```

```
pwad$Australia_Exposed_Shrub_Subsurface_vs_Australia_Exposed_Shrub_Surface
```

```
## Permutation: free
## Number of permutations: 999
##
## Terms added sequentially (first to last)
##
##            Df SumsOfSqs MeanSqs F.Model    R2 Pr(>F)
## allFactors  1    1.3989 1.39892  8.5781 0.488  0.003 **
## Residuals   9    1.4677 0.16308         0.512
## Total      10    2.8666                 1.000
```

```
## ---
## Signif. codes:  0 '***' 0.001 '**' 0.01 '*' 0.05 '.' 0.1 ' ' 1
```

```
pwad$Australia_Exposed_Fringe_Subsurface_vs_Australia_Exposed_Fringe_Surface
```

```
## Permutation: free
## Number of permutations: 999
##
## Terms added sequentially (first to last)
##
##            Df SumsOfSqs MeanSqs F.Model      R2 Pr(>F)
## allFactors  1   0.39084 0.39084  2.7028 0.23095   0.02 *
## Residuals   9   1.30148 0.14461         0.76905
## Total      10   1.69232                 1.00000
## ---
## Signif. codes:  0 '***' 0.001 '**' 0.01 '*' 0.05 '.' 0.1 ' ' 1
```

```
pwad$Australia_Sheltered_Shrub_Surface_vs_Australia_Sheltered_Fringe_Surface
```

```
## Permutation: free
## Number of permutations: 999
##
## Terms added sequentially (first to last)
##
##            Df SumsOfSqs MeanSqs F.Model      R2 Pr(>F)
## allFactors  1    1.8036 1.80362  14.821 0.62218  0.006 **
## Residuals   9    1.0952 0.12169         0.37782
## Total      10    2.8989                 1.00000
## ---
## Signif. codes:  0 '***' 0.001 '**' 0.01 '*' 0.05 '.' 0.1 ' ' 1
```

```
pwad$Australia_Sheltered_Shrub_Surface_vs_Australia_Exposed_Shrub_Surface
```

```
## Permutation: free
## Number of permutations: 999
##
## Terms added sequentially (first to last)
##
##            Df SumsOfSqs MeanSqs F.Model     R2 Pr(>F)
## allFactors  1   0.99829 0.99829  6.8505 0.4613  0.011 *
## Residuals   8   1.16580 0.14573         0.5387
## Total       9   2.16409                 1.0000
## ---
## Signif. codes:  0 '***' 0.001 '**' 0.01 '*' 0.05 '.' 0.1 ' ' 1
```

```
pwad$Australia_Sheltered_Fringe_Surface_vs_Australia_Exposed_Fringe_Surface
```

```
## Permutation: free
## Number of permutations: 999
##
## Terms added sequentially (first to last)
##
##            Df SumsOfSqs MeanSqs F.Model      R2 Pr(>F)
## allFactors  1    1.6989 1.69888  13.536 0.57512  0.003 **
## Residuals  10    1.2551 0.12551         0.42488
## Total      11    2.9540                 1.00000
## ---
```

```
## Signif. codes:  0 '***' 0.001 '**' 0.01 '*' 0.05 '.' 0.1 ' ' 1
```

```r
pwad$Australia_Exposed_Shrub_Surface_vs_Australia_Exposed_Fringe_Surface
```

```
## Permutation: free
## Number of permutations: 999
##
## Terms added sequentially (first to last)
##
##            Df SumsOfSqs MeanSqs F.Model      R2 Pr(>F)
## allFactors  1    1.6822 1.68217  11.421 0.55927  0.004 **
## Residuals   9    1.3256 0.14729         0.44073
## Total      10    3.0078                 1.00000
## ---
## Signif. codes:  0 '***' 0.001 '**' 0.01 '*' 0.05 '.' 0.1 ' ' 1
```

6.2.3 Comparison within Saudi Arabia

```r
pwad$`Saudi Arabia_Sheltered_Shrub_Subsurface_vs_Saudi Arabia_Exposed_Shrub_Subsurface`
```

```
## Permutation: free
## Number of permutations: 999
##
## Terms added sequentially (first to last)
##
##            Df SumsOfSqs MeanSqs F.Model     R2 Pr(>F)
## allFactors  1    1.5454 1.54537  9.9807 0.4162  0.001 ***
## Residuals  14    2.1677 0.15484         0.5838
## Total      15    3.7131                 1.0000
## ---
## Signif. codes:  0 '***' 0.001 '**' 0.01 '*' 0.05 '.' 0.1 ' ' 1
```

```r
pwad$`Saudi Arabia_Sheltered_Shrub_Subsurface_vs_Saudi Arabia_Sheltered_Fringe_Subsurface`
```

```
## Permutation: free
## Number of permutations: 999
##
## Terms added sequentially (first to last)
##
##            Df SumsOfSqs MeanSqs F.Model      R2 Pr(>F)
## allFactors  1    2.7600 2.76003  18.357 0.56733  0.001 ***
## Residuals  14    2.1049 0.15035         0.43267
## Total      15    4.8650                 1.00000
## ---
## Signif. codes:  0 '***' 0.001 '**' 0.01 '*' 0.05 '.' 0.1 ' ' 1
```

```r
pwad$`Saudi Arabia_Exposed_Shrub_Subsurface_vs_Saudi Arabia_Exposed_Fringe_Subsurface`
```

```
## Permutation: free
## Number of permutations: 999
##
## Terms added sequentially (first to last)
##
##            Df SumsOfSqs MeanSqs F.Model      R2 Pr(>F)
## allFactors  1    2.3478 2.34777  12.425 0.43712  0.001 ***
## Residuals  16    3.0233 0.18896         0.56288
## Total      17    5.3711                 1.00000
```

```
## ---
## Signif. codes:  0 '***' 0.001 '**' 0.01 '*' 0.05 '.' 0.1 ' ' 1
```

```r
pwad$`Saudi Arabia_Sheltered_Shrub_Subsurface_vs_Saudi Arabia_Sheltered_Shrub_Surface`
```

```
## Permutation: free
## Number of permutations: 999
##
## Terms added sequentially (first to last)
##
##            Df SumsOfSqs MeanSqs F.Model      R2 Pr(>F)
## allFactors  1    1.4887  1.4887  9.3627 0.40075  0.001 ***
## Residuals  14    2.2260  0.1590         0.59925
## Total      15    3.7146                 1.00000
## ---
## Signif. codes:  0 '***' 0.001 '**' 0.01 '*' 0.05 '.' 0.1 ' ' 1
```

```r
pwad$`Saudi Arabia_Sheltered_Fringe_Subsurface_vs_Saudi Arabia_Exposed_Fringe_Subsurface`
```

```
## Permutation: free
## Number of permutations: 999
##
## Terms added sequentially (first to last)
##
##            Df SumsOfSqs MeanSqs F.Model      R2 Pr(>F)
## allFactors  1    1.0315 1.03147  5.5745 0.25839  0.001 ***
## Residuals  16    2.9605 0.18503         0.74161
## Total      17    3.9920                 1.00000
## ---
## Signif. codes:  0 '***' 0.001 '**' 0.01 '*' 0.05 '.' 0.1 ' ' 1
```

```r
pwad$`Saudi Arabia_Sheltered_Fringe_Subsurface_vs_Saudi Arabia_Sheltered_Fringe_Surface`
```

```
## Permutation: free
## Number of permutations: 999
##
## Terms added sequentially (first to last)
##
##            Df SumsOfSqs MeanSqs F.Model      R2 Pr(>F)
## allFactors  1   0.89953 0.89953  6.5698 0.29109  0.001 ***
## Residuals  16   2.19071 0.13692         0.70891
## Total      17   3.09025                 1.00000
## ---
## Signif. codes:  0 '***' 0.001 '**' 0.01 '*' 0.05 '.' 0.1 ' ' 1
```

```r
pwad$`Saudi Arabia_Exposed_Shrub_Subsurface_vs_Saudi Arabia_Exposed_Shrub_Surface`
```

```
## Permutation: free
## Number of permutations: 999
##
## Terms added sequentially (first to last)
##
##            Df SumsOfSqs MeanSqs F.Model      R2 Pr(>F)
## allFactors  1    1.9316 1.93157  15.227 0.48762  0.001 ***
## Residuals  16    2.0296 0.12685         0.51238
## Total      17    3.9612                 1.00000
## ---
```

```
## Signif. codes:  0 '***' 0.001 '**' 0.01 '*' 0.05 '.' 0.1 ' ' 1
```

```r
pwad$`Saudi Arabia_Exposed_Fringe_Subsurface_vs_Saudi Arabia_Exposed_Fringe_Surface`
```

```
## Permutation: free
## Number of permutations: 999
##
## Terms added sequentially (first to last)
##
##            Df SumsOfSqs MeanSqs F.Model      R2 Pr(>F)
## allFactors  1    0.9082 0.90820  4.6801 0.22631  0.001 ***
## Residuals  16    3.1049 0.19406         0.77369
## Total      17    4.0131                 1.00000
## ---
## Signif. codes:  0 '***' 0.001 '**' 0.01 '*' 0.05 '.' 0.1 ' ' 1
```

```r
pwad$`Saudi Arabia_Sheltered_Shrub_Surface_vs_Saudi Arabia_Sheltered_Fringe_Surface`
```

```
## Permutation: free
## Number of permutations: 999
##
## Terms added sequentially (first to last)
##
##            Df SumsOfSqs MeanSqs F.Model      R2 Pr(>F)
## allFactors  1    3.1942  3.1942  22.108 0.58014  0.001 ***
## Residuals  16    2.3117  0.1445         0.41986
## Total      17    5.5059                 1.00000
## ---
## Signif. codes:  0 '***' 0.001 '**' 0.01 '*' 0.05 '.' 0.1 ' ' 1
```

```r
pwad$`Saudi Arabia_Sheltered_Shrub_Surface_vs_Saudi Arabia_Exposed_Shrub_Surface`
```

```
## Permutation: free
## Number of permutations: 999
##
## Terms added sequentially (first to last)
##
##            Df SumsOfSqs MeanSqs F.Model      R2 Pr(>F)
## allFactors  1    1.3066 1.30658  10.013 0.38492  0.001 ***
## Residuals  16    2.0879 0.13049         0.61508
## Total      17    3.3945                 1.00000
## ---
## Signif. codes:  0 '***' 0.001 '**' 0.01 '*' 0.05 '.' 0.1 ' ' 1
```

```r
pwad$`Saudi Arabia_Sheltered_Fringe_Surface_vs_Saudi Arabia_Exposed_Fringe_Surface`
```

```
## Permutation: free
## Number of permutations: 999
##
## Terms added sequentially (first to last)
##
##            Df SumsOfSqs MeanSqs F.Model      R2 Pr(>F)
## allFactors  1    1.1852 1.18516  8.1207 0.33667  0.001 ***
## Residuals  16    2.3351 0.14594         0.66333
## Total      17    3.5203                 1.00000
## ---
## Signif. codes:  0 '***' 0.001 '**' 0.01 '*' 0.05 '.' 0.1 ' ' 1
```

```
pwad$`Saudi Arabia_Exposed_Shrub_Surface_vs_Saudi Arabia_Exposed_Fringe_Surface`
```

```
## Permutation: free
## Number of permutations: 999
##
## Terms added sequentially (first to last)
##
##            Df SumsOfSqs MeanSqs F.Model      R2 Pr(>F)
## allFactors  1    2.8017 2.80167  21.232 0.57027  0.002 **
## Residuals  16    2.1112 0.13195         0.42973
## Total      17    4.9129                 1.00000
## ---
## Signif. codes:  0 '***' 0.001 '**' 0.01 '*' 0.05 '.' 0.1 ' ' 1
```

6.2.4 PERMANOVA at genus level

A PERMANOVA on the agglomerated tables at genus levels

```
sp_log_gen <- transform_sample_counts(gen, function(x) log(1 + x) )

sp_log_gen_bray <- phyloseq::distance(sp_log_gen, method = "bray")

log_gen_disp_c <- betadisper(sp_log_gen_bray, meta_sp_trimmed$GeographicRegion)
log_gen_disp_e <- betadisper(sp_log_gen_bray, meta_sp_trimmed$Exposure)
log_gen_disp_z <- betadisper(sp_log_gen_bray, meta_sp_trimmed$Zone)
log_gen_disp_d <- betadisper(sp_log_gen_bray, meta_sp_trimmed$Depth)

anova(log_gen_disp_c)
```

```
## Analysis of Variance Table
##
## Response: Distances
##            Df  Sum Sq   Mean Sq F value Pr(>F)
## Groups      1 0.00272 0.0027224  0.4597 0.4992
## Residuals 113 0.66926 0.0059227
```

```
anova(log_gen_disp_e)
```

```
## Analysis of Variance Table
##
## Response: Distances
##            Df  Sum Sq   Mean Sq F value Pr(>F)
## Groups      1 0.00023 0.0002327  0.0359   0.85
## Residuals 113 0.73194 0.0064773
```

```
anova(log_gen_disp_z)
```

```
## Analysis of Variance Table
##
## Response: Distances
##            Df  Sum Sq  Mean Sq F value    Pr(>F)
## Groups      1 0.15532 0.155325  32.286 1.052e-07 ***
## Residuals 113 0.54363 0.004811
## ---
## Signif. codes:  0 '***' 0.001 '**' 0.01 '*' 0.05 '.' 0.1 ' ' 1
```

```r
anova(log_gen_disp_d)
```

```
## Analysis of Variance Table
##
## Response: Distances
##            Df  Sum Sq   Mean Sq F value  Pr(>F)
## Groups      1 0.02132 0.0213152  5.1131 0.02566 *
## Residuals 113 0.47107 0.0041688
## ---
## Signif. codes:  0 '***' 0.001 '**' 0.01 '*' 0.05 '.' 0.1 ' ' 1
```

The p-values indicate an even dispersion of the variance between 3 factors. We therefore proceed with the analysis, keeping in mind that the dispesion is not homogenious between the two levels of the factor Zone.

```r
ad_gen <- adonis2(sp_log_gen_bray~GeographicRegion*Exposure*Zone*Depth,
                  meta_sp_trimmed, permutations = 999,
               strata = NULL, contr.unordered = "contr.sum",
               contr.ordered = "contr.poly")
```

Supplementary Table S10

Summary table showing the results of the PERMANOVA on the abundance table at genus level with significant differences between the interaction factors geographic region, exposure, zone, and depth.

```r
print(ad_gen)
```

```
## Permutation test for adonis under reduced model
## Terms added sequentially (first to last)
## Permutation: free
## Number of permutations: 999
##
## adonis2(formula = sp_log_gen_bray ~ GeographicRegion * Exposure * Zone * Depth, data = meta_sp_trimm
##                                      Df SumOfSqs      R2       F Pr(>F)
## GeographicRegion                      1   2.0686 0.09135 34.6124  0.001
## Exposure                              1   1.2083 0.05336 20.2174  0.001
## Zone                                  1   4.4478 0.19642 74.4202  0.001
## Depth                                 1   3.2580 0.14387 54.5124  0.001
## GeographicRegion:Exposure             1   0.8298 0.03664 13.8840  0.001
## GeographicRegion:Zone                 1   0.9619 0.04248 16.0943  0.001
## Exposure:Zone                         1   0.5891 0.02602  9.8575  0.001
## GeographicRegion:Depth                1   0.5969 0.02636  9.9874  0.001
## Exposure:Depth                        1   0.2134 0.00942  3.5710  0.004
## Zone:Depth                            1   1.0686 0.04719 17.8794  0.001
## GeographicRegion:Exposure:Zone        1   0.6203 0.02739 10.3790  0.001
## GeographicRegion:Exposure:Depth       1   0.1656 0.00731  2.7713  0.006
## GeographicRegion:Zone:Depth           1   0.3069 0.01355  5.1345  0.001
## Exposure:Zone:Depth                   1   0.1540 0.00680  2.5760  0.013
## GeographicRegion:Exposure:Zone:Depth  1   0.2385 0.01053  3.9903  0.001
## Residual                             99   5.9168 0.26129
## Total                               114  22.6444 1.00000
##
## GeographicRegion                     ***
## Exposure                             ***
## Zone                                 ***
## Depth                                ***
```

```
## GeographicRegion:Exposure            ***
## GeographicRegion:Zone                ***
## Exposure:Zone                        ***
## GeographicRegion:Depth               ***
## Exposure:Depth                       **
## Zone:Depth                           ***
## GeographicRegion:Exposure:Zone       ***
## GeographicRegion:Exposure:Depth      **
## GeographicRegion:Zone:Depth          ***
## Exposure:Zone:Depth                  *
## GeographicRegion:Exposure:Zone:Depth ***
## Residual
## Total
## ---
## Signif. codes:  0 '***' 0.001 '**' 0.01 '*' 0.05 '.' 0.1 ' ' 1
```

7 Network analysis

The networks were created in Cytoscape and visualised in Gephi. The plug-in Centiscape calculated network parameters which can be exported to .CSV files and used for statistical analysis and visualisation in R.

```
edge_tab <- read.csv("~/Desktop/SpatialPatterns/sp_data/sp_data_tables/Statistic_Network_edge.csv",
                     stringsAsFactors = F)
node_tab <- read.csv("~/Desktop/SpatialPatterns/sp_data/sp_data_tables/Statistic_Network_node.csv",
                     stringsAsFactors = F)
```

Use this code to select the most abundant phyla and group the less abundant phyla as "Other". To do so, we calculate the frequency of each category in the table and Create a new column and replace low-frequency phyla for "Other". We can then save it into a file and repeat with the edge table.

```
phyla_freq <- sort(table(node_tab$phylum), decreasing = T)
low_phyla <- names(phyla_freq[13:length(phyla_freq)])

node_tab$phylum_top<-node_tab$phylum
node_tab[node_tab$phylum_top%in%low_phyla, "phylum_top"]<-"Other"

node_tab$Depth <- ordered(node_tab$Depth, levels = c("Surface", "Subsurface"))
edge_tab$Depth <- ordered(edge_tab$Depth, levels = c("Surface", "Subsurface"))

row.names(node_tab)<-NULL
write.csv(node_tab,
          file = "~/Desktop/SpatialPatterns/sp_data/sp_data_tables/Statistic_Network_node_topPhyla.csv"
          quote = F, row.names = F)
```

```
node_tab$phylum_top<- factor(node_tab$phylum_top, levels = c("Acidobacteria","Actinobacteria", "Bactero
                                          "Chloroflexi", "Cyanobacteria", "Gemmatimodetes",
                                          "Nitrospirae", "Planctomycetes", "Proteobacteria",
                                          "Spirochaetes", "Zixibacteria", "Other"))
```

7.1 Network parameters

This plots the different topological metrics as violin plots across all different factors.

```
eb <- ggplot(edge_tab, aes(Zone, EdgeBetweenness_unDir, fill = Depth))+
  geom_violin()+
```

```r
  scale_fill_brewer(palette = "Paired")+
  labs(title = "Edge Betweenness")+
  facet_grid(Exposure~GeographicRegion)+
  theme_classic()+
  theme(axis.title = element_blank(),
        legend.position = "none")+
  scale_y_log10()


be <- ggplot(node_tab, aes(Zone, Betweenness.unDir + 1, fill = Depth))+
  geom_violin()+
  scale_fill_brewer(palette = "Paired")+
  labs(title = "Betweenness")+
  facet_grid(Exposure~GeographicRegion)+
  theme_classic()+
  theme(axis.title = element_blank(),
        legend.position = "none")+
  scale_y_log10()


br <- ggplot(node_tab, aes(Zone, Bridging.unDir + 1, fill = Depth))+
  geom_violin()+
  scale_fill_brewer(palette = "Paired")+
  labs(title = "Bridging")+
  facet_grid(Exposure~GeographicRegion)+
  theme_classic()+
  theme(axis.title = element_blank(),
        legend.position = "none")+
  scale_y_log10()


cl <- ggplot(node_tab, aes(Zone, Closeness.unDir, fill = Depth))+
  geom_violin()+
  scale_fill_brewer(palette = "Paired")+
  labs(title = "Closeness")+
  facet_grid(Exposure~GeographicRegion)+
  theme_classic()+
  theme(axis.title = element_blank(),
        legend.position = "none")+
  scale_y_log10()


cc <-ggplot(node_tab, aes(Zone, ClusteringCoefficient, fill = Depth))+
  geom_violin()+
  scale_fill_brewer(palette = "Paired")+
  labs(title = "Clustering Coefficient")+
  facet_grid(Exposure~GeographicRegion)+
  theme(axis.title = element_blank(),
        legend.position = "none")+
  theme_classic()


ec <- ggplot(node_tab, aes(Zone, Eccentricity.unDir, fill = Depth))+
```

```r
  geom_violin()+
  scale_fill_brewer(palette = "Paired")+
  labs(title = "Eccentricity")+
  facet_grid(Exposure~GeographicRegion)+
  theme_classic()+
  theme(axis.title = element_blank(),
        legend.position = "none")+
  scale_y_log10()


ev <- ggplot(node_tab, aes(Zone, EigenVector.unDir, fill = Depth))+
  geom_violin()+
  scale_fill_brewer(palette = "Paired")+
  labs(title = "Eigen Vector")+
  facet_grid(Exposure~GeographicRegion)+
  theme(axis.title = element_blank(),
        legend.position = "none")+
  theme_classic()


rd <- ggplot(node_tab, aes(Zone, Radiality.unDir, fill = Depth))+
  geom_violin()+
  scale_fill_brewer(palette = "Paired")+
  labs(title = "Radiality")+
  facet_grid(Exposure~GeographicRegion)+
  theme(axis.title = element_blank(),
        legend.position = "none")+
  theme_classic()


st <- ggplot(node_tab, aes(Zone, Stress.unDir + 1, fill = Depth))+
  geom_violin()+
  scale_fill_brewer(palette = "Paired")+
  labs(title = "Stress")+
  facet_grid(Exposure~GeographicRegion)+
  theme_classic()+
  theme(axis.title = element_blank(),
        legend.position = "none")+
  scale_y_log10()


tp <- ggplot(node_tab, aes(Zone, TopologicalCoefficient, fill = Depth))+
  geom_violin()+
  scale_fill_brewer(palette = "Paired")+
  labs(title = "Topological Coefficient")+
  facet_grid(Exposure~GeographicRegion)+
  theme(axis.title = element_blank(),
        legend.position = "none")+
  theme_classic()


grid <- ggarrange(eb,be,br,cl,cc,ec,ev,rd,st,tp, ncol = 5, nrow = 2,
                  common.legend = T, legend = "bottom")
```

7.2 Node metrics by phylum

```
coul <- brewer.pal(12, "Paired")
coul <- colorRampPalette(coul)(13)
names(coul) <- levels(node_tab$phylum_top)
colourScaleF <- scale_fill_manual(name = "Phylum", values = coul)
colourScaleC <- scale_colour_manual(name = "Phylum", values = coul)
```

7.2.1 Betweenness

```
bet <- ggplot(node_tab, aes(phylum_top, fill = phylum_top))+
  stat_summary(aes(y = Betweenness.unDir + 1), fun.y = "mean", geom = "bar")+
  stat_summary(aes(y = Betweenness.unDir +1), fun.data = "mean_se",
               geom = "errorbar", width = .2)

bet +
  facet_grid(cols = vars(GeographicRegion, Zone), rows = vars(Exposure, Depth))+
  scale_y_log10()+
  guides(fill = guide_legend(title = "Phylum", ncol = 1), colour = FALSE)+
  labs(y = paste("log(Betweenness+1)"))+
  scale_fill_manual(values =  c("#C8E6C9", "#52AF43", "#1B5E20",
                                "#B89B74", "#B15928", "#ED4F50",
                                 "#FDA440", "#F8F18F", "#B294C7",
                                "#2A7FB7", "#FFCCBC", "#80DEEA", "#BDBDBD"))+
  theme_classic()+
  theme(axis.title.x = element_blank(),
        axis.text.x = element_blank(),
        axis.ticks.x = element_blank(),
        )
```

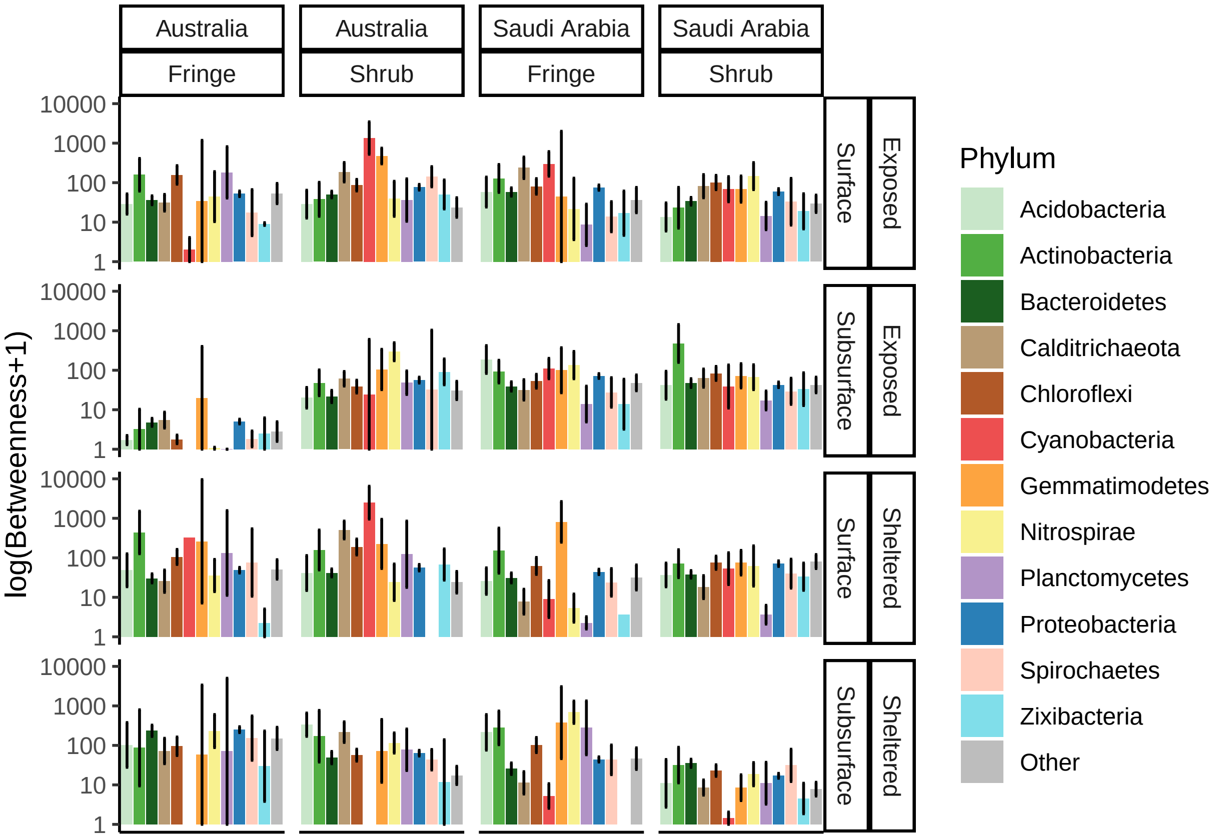

Figure 3A

Betweenness centrality of the 12 most prevalent phyla (within the networks). The remaining phyla were grouped as 'Other'. Mean scores are displayed with standard errors calculated by the stat_summary function in ggplot2. Bars without standard errors display single occurrences.

7.2.2 Topological coefficiant

```
top <- ggplot(node_tab, aes(phylum_top, fill = phylum_top))+
  stat_summary(aes(y = TopologicalCoefficient), fun.y = "mean", geom = "bar")+
  stat_summary(aes(y = TopologicalCoefficient), fun.data = "mean_se",
               geom = "errorbar", width = .2)

top +
  facet_grid(cols = vars(GeographicRegion, Zone), rows = vars(Exposure, Depth))+
  labs(y = "Topological Coefficient")+
  scale_fill_manual(values =  c("#C8E6C9", "#52AF43", "#1B5E20",
                                "#B89B74", "#B15928", "#ED4F50",
                                "#FDA440", "#F8F18F", "#B294C7",
                                "#2A7FB7", "#FFCCBC", "#80DEEA", "#BDBDBD"))+
  theme_classic(base_size = 20)+
  theme(axis.title.x = element_blank(),
        axis.text.x = element_blank(),
        axis.ticks.x = element_blank(),
        legend.position = "none"
        )
```

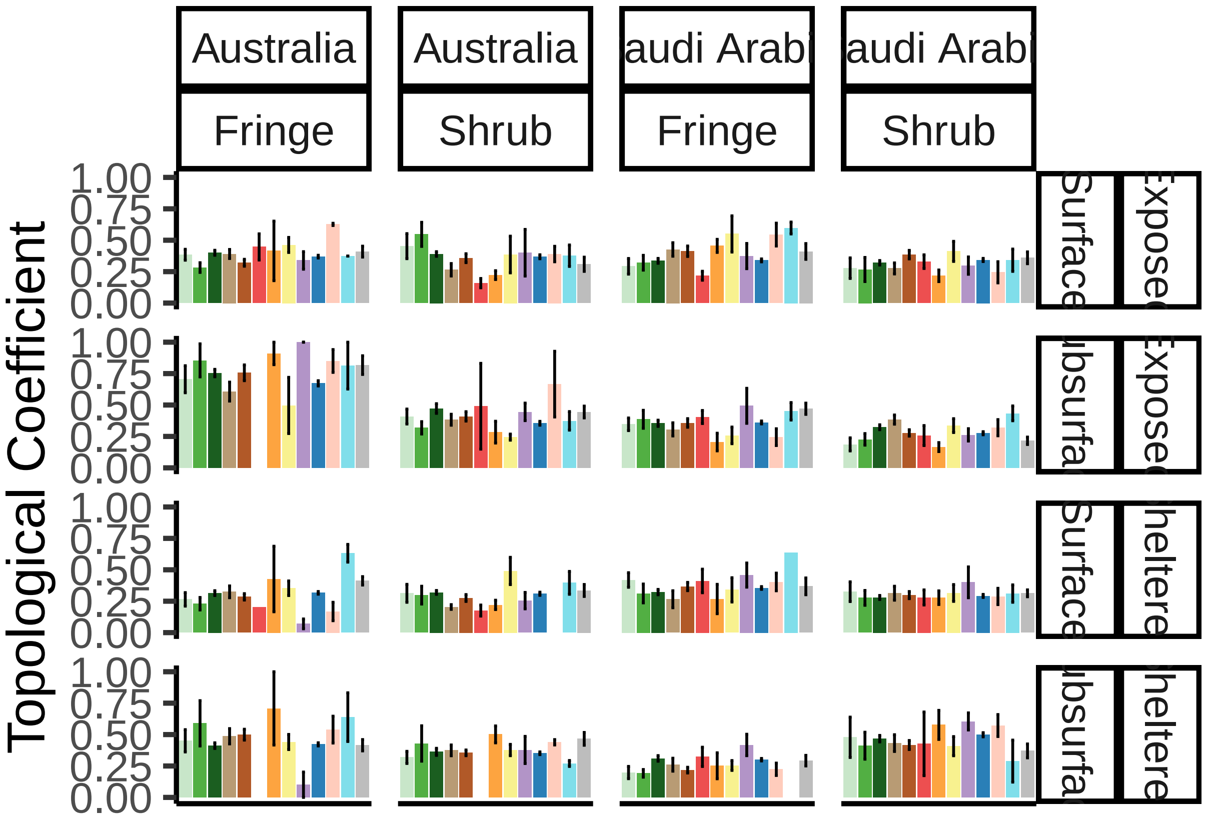

Figure 3B

Topological coefficient of the 12 most prevalent phyla (within the networks). The remaining phyla were grouped as 'Other'. Mean scores are displayed with standard errors calculated by the stat_summary function in ggplot2. Bars without standard errors display single occurrences.

7.3 Relative importance of nodes within the networks

This command normalizes the degree by the number of nodes in each network.

```
node_tab_af <- unite(node_tab, AllFactors, GeographicRegion, Exposure, Zone, Depth,
                     sep = " ", remove = F)

node_tab_norm <- node_tab_af %>%
  group_by(AllFactors) %>%
  mutate(degree_norm = normalize(Degree.unDir, method = "standardize"))
```

7.3.1 Keystone species plot

The log(Betweenness centrality) plotted against normalized degree score. The further in the node is placed in the top righthand corner, the more important it is for the connectivity of the network.

```
ggplot(node_tab_norm, aes(degree_norm, log10(Betweenness.unDir), colour = phylum_top))+
  geom_point()+
  labs(x = "Normalized Degree", y = "log(Betweenness centrality)")+
  facet_grid(cols = vars(GeographicRegion, Zone), rows = vars(Exposure, Depth))+
  scale_colour_manual(values =  c("#C8E6C9", "#52AF43", "#1B5E20",
                                  "#B89B74", "#B15928", "#ED4F50",
                                   "#FDA440", "#F8F18F", "#B294C7",
```

```r
                                 "#2A7FB7", "#FFCCBC", "#80DEEA", "#BDBDBD"))+
  theme_bw()
```

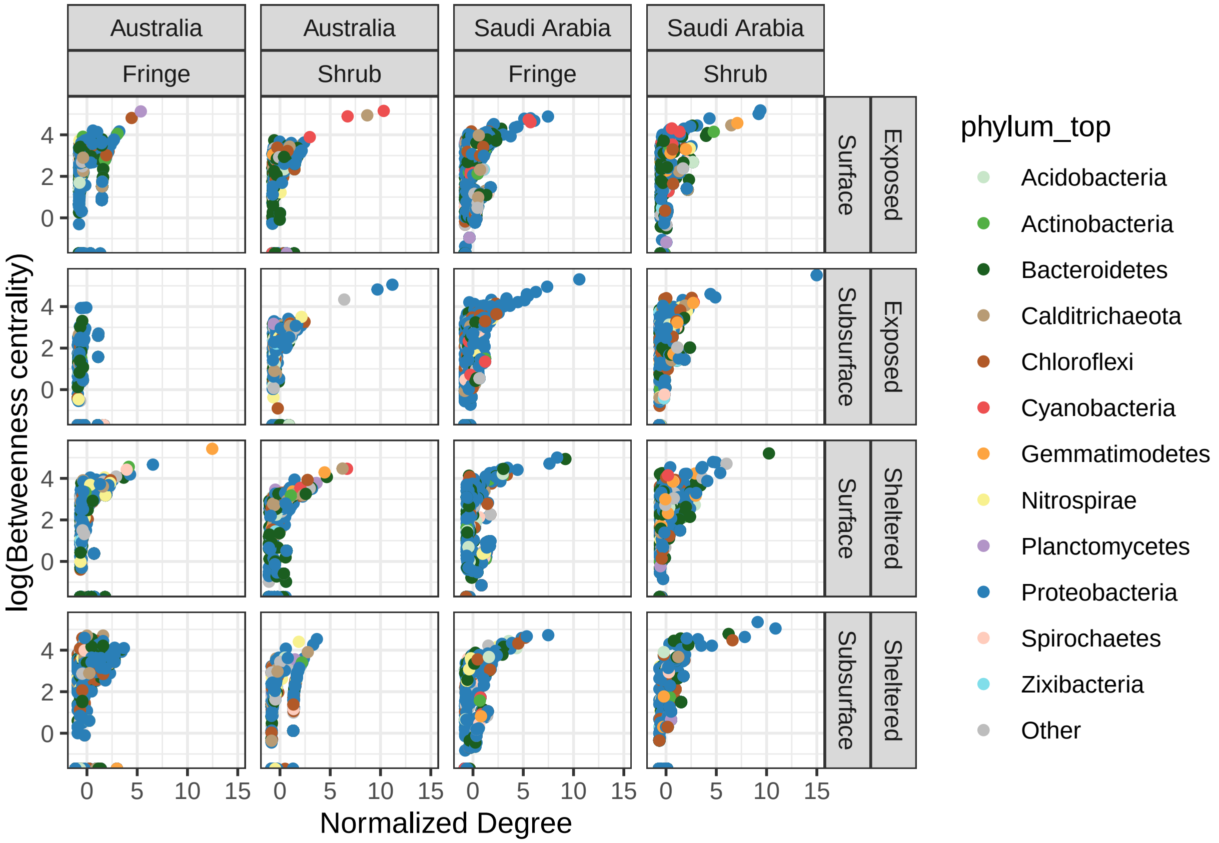

Supplementary Figure S9

The betweenness centrality scores plotted against the normalized node degree to indicate 'keystone species' as important connectors within co-occurrence networks.

7.3.2 Proportion of nodes by node degree

Kernel density estimation of the relative abundance of nodes per normalized node degree.

```r
ggplot(node_tab_norm, aes(degree_norm, colour = phylum_top, fill = phylum_top))+
  geom_density(position = "fill")+
  labs(x = "Normalized node degree", y = "Relative proportion of nodes")+
  facet_grid(cols = vars(GeographicRegion, Zone), rows = vars(Exposure, Depth))+
  scale_colour_manual(values =  c("#C8E6C9", "#52AF43", "#1B5E20",
                                 "#B89B74", "#B15928", "#ED4F50",
                                  "#FDA440", "#F8F18F", "#B294C7",
                                 "#2A7FB7", "#FFCCBC", "#80DEEA", "#BDBDBD"))+
  scale_fill_manual(values =  c("#C8E6C9", "#52AF43", "#1B5E20",
                                 "#B89B74", "#B15928", "#ED4F50",
                                  "#FDA440", "#F8F18F", "#B294C7",
                                 "#2A7FB7", "#FFCCBC", "#80DEEA", "#BDBDBD"))+
  guides(fill = guide_legend(title = "Phylum"), colour = guide_legend(title = "Phylum"))+
  theme_classic()+
  theme(legend.position = "bottom",
```

```
        strip.text.y = element_text(size = 7),
        legend.key.width = unit(0.5, 'cm'))
```

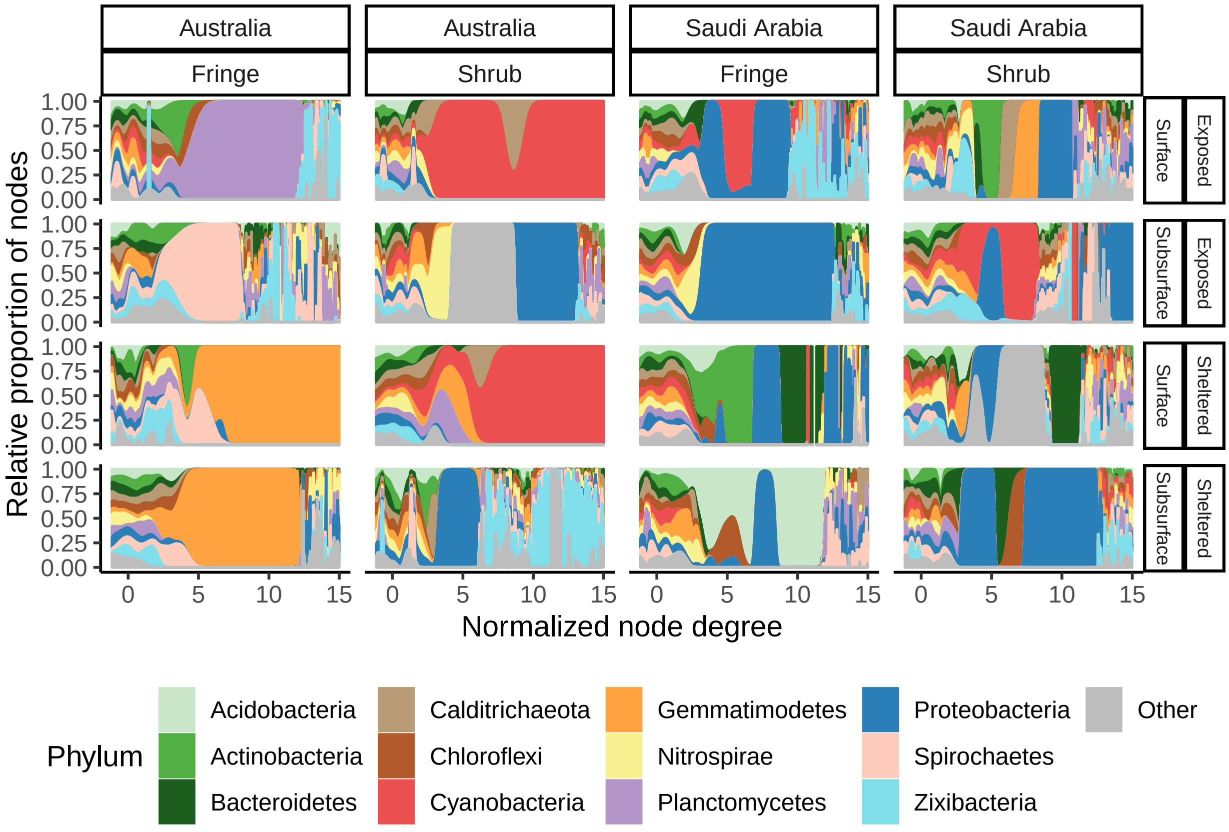

Figure 4

Dendity plots of the relative proportion of nodes against the normalized node degree across the experimental factors, coloured by phylum. The proportion of nodes is calculated by the Kernel-Density function.

8. Fuctional assignments

8.1 FAPROTAX v 1.2.1

FAPROTAX uses a python based script to assign functions to the taxonomy of our abundance table. We need to modify the abundance table and join it with the taxonomy table into the format which FAPROTAX requires. First, we transpose the abundance table so that the rows are ASVs, after which we join it to the taxonomy table. The taxonomic information needs to be in a single column at the end of the table, which is waht we do with the 'unite()' command from the 'tidyr' package. The resulting data.frame is written into a tab seperated file to be processed by the FAPROTAX script. The command used in the terminal was: ./collapse_table.py -i path_to_modified_asv_tax_file.tsv -g path_to_FAPROTAX_database.txt -f -o path_to_folder_where_output_will_be_saved/name_of_output_file.tsv -r path_to_folder_where_output_will_be_saved/ -c "#" -d "taxonomy" -v –omit_columns 0 –column_names_are_in last_comment_line

Note: I had to add a '#' to the first row of the data frame to specify it as a header. This, as well as the name of the taxonomy column can be specified in the 'collapse_table.py' command in python.

```
asv_sp_rar_t <- t(asv_sp_rar)
asv_tax_fap <- cbind(asv_sp_rar_t, tax_sp_rar)
fap_new_taxcol <- unite(asv_tax_fap, taxonomy, Kingdom, Phylum,
```

```
                         Class, Order, Family, Genus, sep = ";", remove = TRUE)
fap_new_taxcol <-as.data.frame(fap_new_taxcol)

write.table(fap_new_taxcol, "functional_assignments/FAPROTAX/asv_tax_FAP.tsv",
            sep = "\t", quote = F)
```

Read the file created by FAPROTAX. Next we delete the rows containing unassigned functions.

```
func_tab <- read.csv("functional_assignments/FAPROTAX/FAPROTAX_sp_out/functional_asv_table.tsv",
                     h = T, fill = T, sep = "\t", row.names = 1, check.names = F)

func_reord <- func_tab[order(rowSums(func_tab), decreasing = T),]

func_reord_nozero <- as.matrix(func_reord[-c(41:90),])
func_reord_nozero_nocont <- func_reord_nozero[,-c(31,74)]
func_tab_t <- as.data.frame(t(func_reord_nozero_nocont))
```

Check for collinearity and prevalence of functions. FAPROTAX supplies a number of redundant functions which often show high collinearity. All functions above a colinearity of 80 % were removed as well as those with a prevalence of appearing in <= 3 sites?

```
func_sp_cor <- cor(func_tab_t, method = "spearman")
func_sp_corr_rem <- func_tab_t[,-c(2,4,5,10,20,21,24,26,30,31,34,36,37,38,39,40)]
```

8.1.1 PERMANOVA of functional assignments

```
func_sp_log <- log(func_sp_corr_rem + 1)

func_sp_log_bray <- vegdist(func_sp_log, method = "bray")

func_log_disp_c <- betadisper(func_sp_log_bray, meta_sp_trimmed$GeographicRegion)
func_log_disp_e <- betadisper(func_sp_log_bray, meta_sp_trimmed$Exposure)
func_log_disp_z <- betadisper(func_sp_log_bray, meta_sp_trimmed$Zone)
func_log_disp_d <- betadisper(func_sp_log_bray, meta_sp_trimmed$Depth)
anova(func_log_disp_c)
```

```
## Analysis of Variance Table
##
## Response: Distances
##            Df  Sum Sq Mean Sq F value   Pr(>F)
## Groups      1 0.06881 0.06881  10.505 0.001565 **
## Residuals 113 0.74019 0.00655
## ---
## Signif. codes:  0 '***' 0.001 '**' 0.01 '*' 0.05 '.' 0.1 ' ' 1
```

```
anova(func_log_disp_e)
```

```
## Analysis of Variance Table
##
## Response: Distances
##            Df  Sum Sq   Mean Sq F value Pr(>F)
## Groups      1 0.00751 0.0075106  0.9329 0.3362
## Residuals 113 0.90971 0.0080506
```

```
anova(func_log_disp_z)
```

```
## Analysis of Variance Table
```

```
##
## Response: Distances
##            Df  Sum Sq   Mean Sq F value Pr(>F)
## Groups      1 0.01877 0.0187749  2.6765 0.1046
## Residuals 113 0.79266 0.0070147
```

```r
anova(func_log_disp_d)
```

```
## Analysis of Variance Table
##
## Response: Distances
##            Df  Sum Sq  Mean Sq F value    Pr(>F)
## Groups      1 0.16390 0.163902  33.889 5.561e-08 ***
## Residuals 113 0.54652 0.004836
## ---
## Signif. codes:  0 '***' 0.001 '**' 0.01 '*' 0.05 '.' 0.1 ' ' 1
```

```r
func_ad <- adonis2(func_sp_log_bray~GeographicRegion*Exposure*Zone*Depth,
                   meta_sp_trimmed, permutations = 999,
                   strata = NULL, contr.unordered = "contr.sum",
                   contr.ordered = "contr.poly")
```

Supplementary Table S11

Summary table showing the results of the PERMANOVA on the functional assignments (FAPROTAX) with significant differences between the interaction factors geographic region, exposure, zone, and depth.

```r
func_ad
```

```
## Permutation test for adonis under reduced model
## Terms added sequentially (first to last)
## Permutation: free
## Number of permutations: 999
##
## adonis2(formula = func_sp_log_bray ~ GeographicRegion * Exposure * Zone * Depth, data = meta_sp_trim
##                                        Df SumOfSqs      R2       F Pr(>F)
## GeographicRegion                        1   1.0219 0.12004 39.3432  0.001
## Exposure                                1   0.3782 0.04443 14.5622  0.001
## Zone                                    1   0.4549 0.05344 17.5155  0.001
## Depth                                   1   0.9090 0.10677 34.9954  0.001
## GeographicRegion:Exposure               1   0.6358 0.07469 24.4794  0.001
## GeographicRegion:Zone                   1   0.6620 0.07776 25.4858  0.001
## Exposure:Zone                           1   0.2943 0.03457 11.3297  0.001
## GeographicRegion:Depth                  1   0.2382 0.02798  9.1721  0.001
## Exposure:Depth                          1   0.1015 0.01193  3.9088  0.001
## Zone:Depth                              1   0.7552 0.08872 29.0772  0.001
## GeographicRegion:Exposure:Zone          1   0.0720 0.00845  2.7711  0.022
## GeographicRegion:Exposure:Depth         1   0.0544 0.00639  2.0947  0.064
## GeographicRegion:Zone:Depth             1   0.1137 0.01336  4.3789  0.003
## Exposure:Zone:Depth                     1   0.0999 0.01173  3.8456  0.003
## GeographicRegion:Exposure:Zone:Depth    1   0.1504 0.01767  5.7921  0.001
## Residual                               99   2.5714 0.30206
## Total                                 114   8.5129 1.00000
##
## GeographicRegion                       ***
## Exposure                               ***
```

```
## Zone                                   ***
## Depth                                  ***
## GeographicRegion:Exposure              ***
## GeographicRegion:Zone                  ***
## Exposure:Zone                          ***
## GeographicRegion:Depth                 ***
## Exposure:Depth                         ***
## Zone:Depth                             ***
## GeographicRegion:Exposure:Zone         *
## GeographicRegion:Exposure:Depth        .
## GeographicRegion:Zone:Depth            **
## Exposure:Zone:Depth                    **
## GeographicRegion:Exposure:Zone:Depth ***
## Residual
## Total
## ---
## Signif. codes:  0 '***' 0.001 '**' 0.01 '*' 0.05 '.' 0.1 ' ' 1
```

The factors Geographic Region and exposure are highly heterogeniouslly dispersed, making the results of the PERMANOVA less trustworthy.

8.1.2 NMDS plot of functional assignments, overlaid with the functions driving the observed patterns

```
func_nmds <- metaMDS(func_sp_corr_rem, distance = "bray", k = 2, trymax = 50)
```

Check stress.

```
print(func_nmds$stress)

func_NMDS=data.frame(x = func_nmds$point[,1], y = func_nmds$point[,2],
                     GeographicRegion  = as.factor(meta_sp_trimmed[,1]),
                     Exposure = as.factor(meta_sp_trimmed[,3]),
                     Zone = as.factor(meta_sp_trimmed[,4]),
                     Depth = as.factor(meta_sp_trimmed[,5]))



func_fit <- envfit(func_nmds, func_sp_corr_rem)
func_vec <- func_fit$vectors
func_r2 <- sort(func_vec$r, decreasing = T)
top_func_r2 <- func_r2[1:13]

func_vecs <- as.data.frame(scores(func_fit, display = "vectors"))
func_vecs <- cbind(func_vecs, Species = rownames(func_vecs))

func_NMDS <- unite(func_NMDS, shape_fill, Zone, Depth, remove = F)

q <- ggplot(func_NMDS)+
  geom_point(mapping = aes(x, y, colour = GeographicRegion,
                          shape = shape_fill, size = Exposure))+
  scale_shape_manual(values = c(1, 16, 2, 17),
                     name = "Zone and Depth",
                     labels = c("Fringe Subsurface",
                                "Fringe Surface",
                                "Shrub Subsurface",
```

```
                                  "Shrub Surface"))+
  geom_segment(data = func_vecs,
               aes(x = 0, xend = NMDS1, y = 0, yend = NMDS2),
               arrow = arrow(length = unit(0.25, "cm")), colour = "grey") +
  geom_text(data = func_vecs, aes(x = NMDS1, y = NMDS2, label = Species),
            size = 3)+
  guides(colour = guide_legend(order = 1),
         size = guide_legend(order = 2),
         shape = guide_legend(order = 3))+
  labs(title = "NMDS plot of functional predictions between Factors", subtitle = "By FAPROTAX",
       x = "NMDS 1", y = "NMDS 2")+
  theme_classic()+
  annotate("text", x = .4, y = -.6, label = "Stress = 0.20")
```

8.1.3 Heat map of the functional assignments across factors

```
func_meta <- cbind(meta_sp_trimmed, func_sp_corr_rem)
func_sp_long <- data.table::melt(setDT(func_meta),
                                 id.vars = c("GeographicRegion", "Exposure", "Zone", "Depth"),
                     measure.vars = 7:30, value.name = "Sample")
names(func_sp_long)[5] <- "Function"
func_sp_long <- unite(func_sp_long, ZoneDepth, Zone, Depth, remove = F, sep = " ")
func_sp_long$ZoneDepth <- as.factor(func_sp_long$ZoneDepth)
func_sp_long$ZoneDepth <- factor(func_sp_long$ZoneDepth,
                                  levels = c("Fringe Surface", "Fringe Subsurface",
                                             "Shrub Surface", "Shrub Subsurface"))



func_sp_long$logSample <- log(func_sp_long$Sample+1)

ggplot(func_sp_long, aes(ZoneDepth, Function, fill = logSample))+
  geom_tile()+
  labs(y = "Assigned Function", fill = "log(Abundance + 1)")+
  scale_fill_viridis_c(option = "C") +
  facet_grid(cols = vars(GeographicRegion), rows = vars(Exposure))+
  theme_classic()+
  theme(axis.title.x = element_blank(), axis.text.x = element_text(angle = 60, hjust = 1),
        axis.text.y = element_text(size = 4),
        legend.position = "top",
  )
```

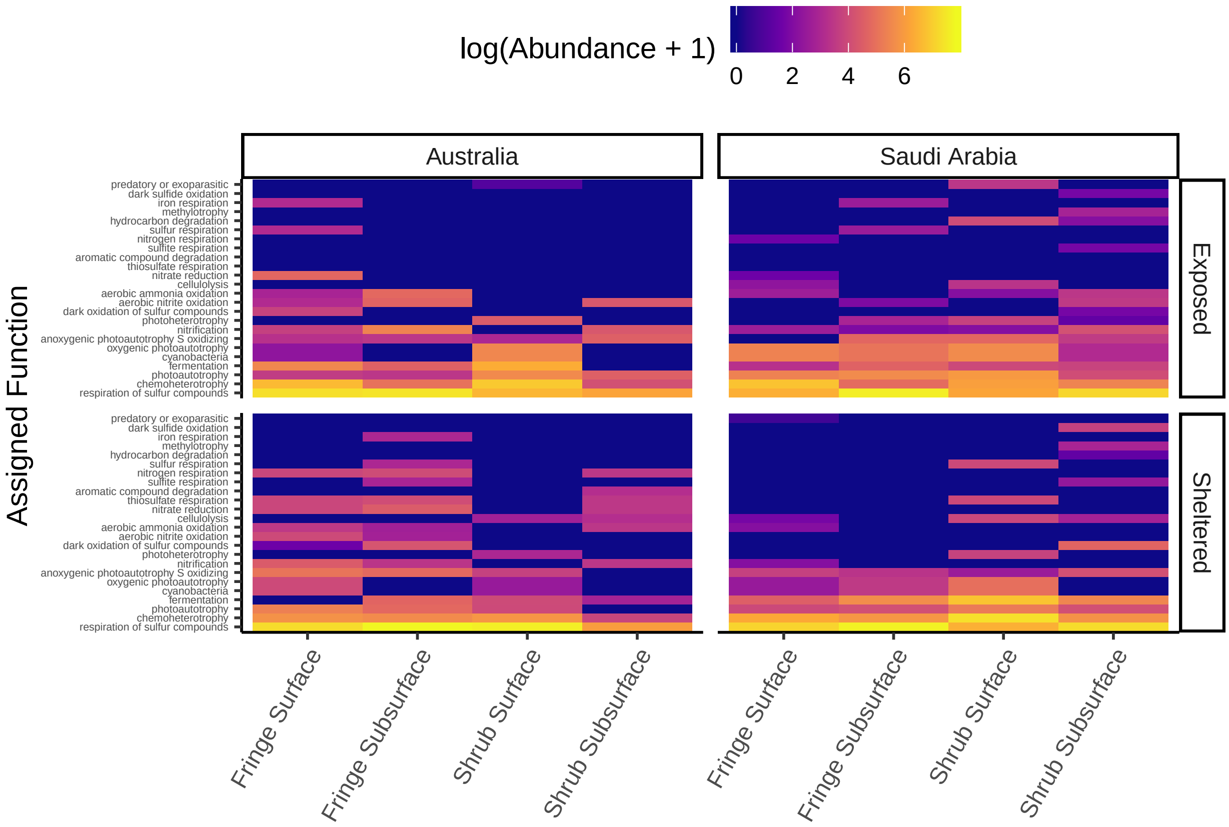

Figure 5

Heat map showing the distributions of bacterial functions that were assigned by FAPROTAX across the experimental factors. The values were log-transformedwith lighter values indicating higher abundances.

Supplementary File 2

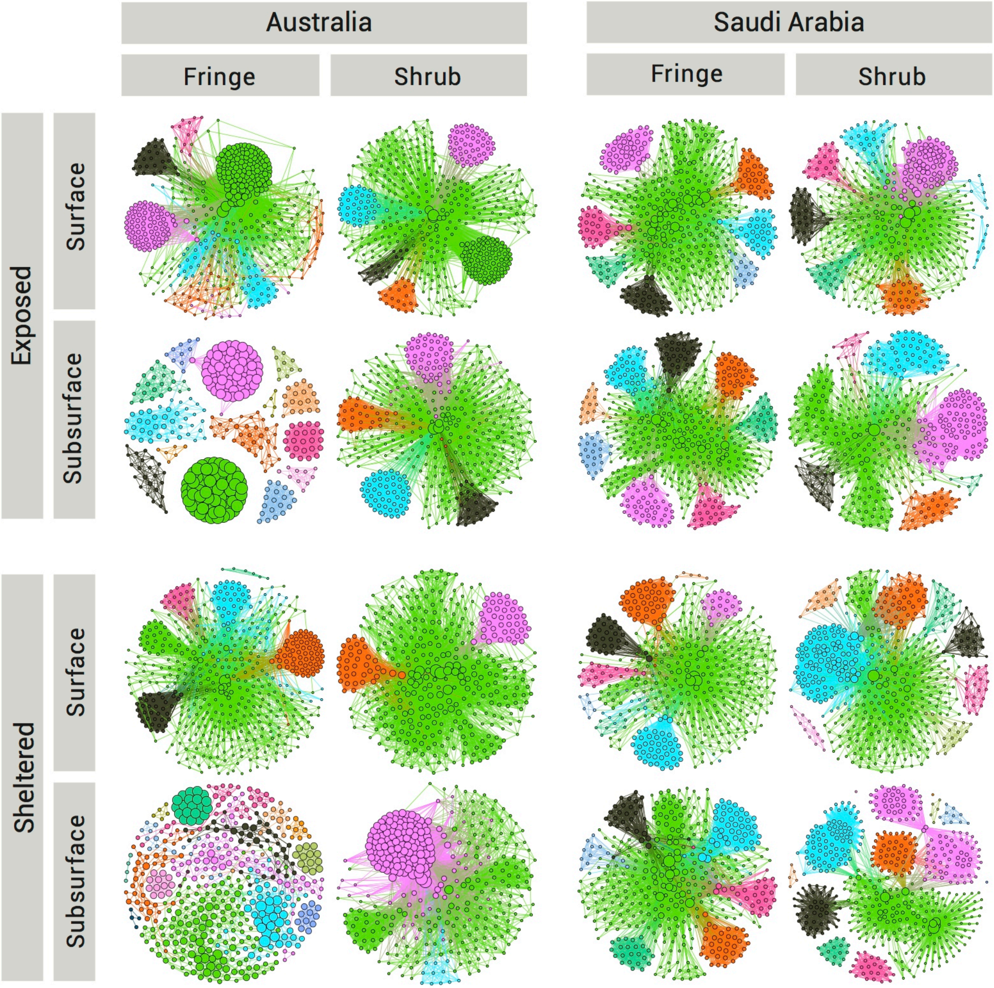

Bacterial co-occurrence networks of each site in Australia and Saudi Arabia across the factors exposure, zone, and depth

The colours denote different modules within each network but do not show connections between networks per se. The size of the nodes is relative to their node degree.
